# Supplementary material for: Euglena’s atypical respiratory chain adapts to the discoidal cristae and flexible metabolism
Source: Nat Commun. 2024 Feb 22;15:1628. doi: 10.1038/s41467-024-46018-z (PMC10884005; doi:10.1038/s41467-024-46018-z)
Supplement: Supplementary file 1 — Supplementary Information [file 41467_2024_46018_MOESM1_ESM.pdf]

Euglena's Atypical Respiratory Chain Adapts to the Discoidal Cristae and Flexible Metabolism

Zhaoxiang He[1]†, Mengchen Wu[1]†, Hongtao Tian[1]†, Liangdong Wang[2], Yiqi Hu[1], Fangzhu Han[1], Jiancang Zhou[3]*, Yong Wang[2,4]*, Long Zhou[1]*

Affiliations:

[1]Department of Biophysics and Department of Critical Care Medicine of Sir Run Run Shaw Hospital, Zhejiang University School of Medicine, Hangzhou 310058, China

[2]College of Life Sciences, Zhejiang University, Hangzhou 310058, China.

[3]Department of Critical Care Medicine, Sir Run Run Shaw Hospital, Zhejiang University School of Medicine, Hangzhou 310016, China

[4]The Provincial International Science and Technology Cooperation Base on Engineering Biology, International Campus of Zhejiang University, Haining, 314400, China

*Corresponding authors. Email: jiancangzhou@zju.edu.cn, yongwang_isb@zju.edu.cn, longzhou@zju.edu.cn

†These authors contributed equally to this work

Supplementary Information

<u>Table of Contents</u>

Supplementary Discussion

Supplementary References

Supplementary Figures 1 to 21

Supplementary Tables 1-9

Supplementary Discussion

The FAS domain subunits

Sequence alignments of MDR-like subunits in euglenozoan CI's FAS domains with MDR-like TERs from *Candida tropicalis* (PDB 1GUF, 4WAS)[1] and human (PDB 2VCY)[1] reveal the lack of a NADPH-recognizing RXR motif in both NDUEG3 and NDUEG5, but a NADH-contacting GXGXXG motif as in the equine alcohol dehydrogenase (ADH) (PDB 1HET) is partially conserved in NDUEG5 with the second glycine replaced by an alanine[2,3]

(Supplementary Figs. 14a,b and 15a-d). A soluble version of *E. gracilis* mitochondrial TER (UniProt: Q5EU90), belonging to the short-chain dehydrogenase/reductase (SDR) superfamily adopting a different fold than MDRs, has been identified and purified[4], but its knockdown fails to influence anaerobic wax production[5]. This leads to the proposal that *E. gracilis* uses ACD instead of TER for FAS while MDR-like TER has never been found for *E. gracilis*[6]. Subunit NDUEG2 has an incomplete CT domain compared to soluble PEPCK structures (Supplementary Fig. 15e)[7,8]. This is similar to another FAS subunit NDUEG1/NDUTT2, which is structurally homologous to the NT substrate binding domain of AMP-forming acyl-CoA synthetase (PDB 3R44) but lacks the CT catalytic domain[9,10] (Supplementary Fig. 15f). Unlike NDUEG1/NDUTT2 which is present in both Alveolata and Discoba (former part of Excavata clade)[11], addition of TER- or PEPCK-like subunits to CI should happen after the branching of euglenozoans from other eukaryotes. Their initial attachments to euglenozoan CI could grant the evolutionary advantage of clustering the rate-limiting FAS enzymes with the electron source of anaerobic ETC at the mitochondrial cristae (see Discussion), but their present functions in Eg-CI await more in-depth knockout investigation.

Structural details of the interactions between the FAS domain and Eg-CI PA

In addition to the strengthening effects from NDUFA6 and NDUFS4, the FAS subunits also associate with Eg-CI PA via their own extensions. One example is the CT vertical helix of NDUEG3 which is clamped between NDUFA6 and NDUFS1B mentioned before (Fig. 2h). Although lacking the catalytic CT domain, Eg-NDUEG1 is structurally more complete than Tt-NDUTT2[9,11] (Supplementary Fig. 15f,g). The elongated NT of Eg-$\alpha 9^{NDUEG1}$, not present in Tt-NDUTT2, polarly contacts an Eg-specific CT helix of NDUFS6, indicating that NDUEG1's extra region serves at least an assembly role (Supplementary Fig. 15f,g). The PEPCK-like subunit NDUEG2 is located on top of core subunit NDUFS1B and is also connected to the above described NDUEG5 subunit by a small, four-helical FAS subunit NDUEG12 (Fig. 2a). It has a ~66 residues NT extension not found in soluble PEPCK, which winds among NDUFS1B, NDUFS4 and NDUEG12 [7,8] (Supplementary Fig. 15e,h). It also has an elongated β8-9 connection (numbered as in PDB 1II2) that forms a double helix bundle with NDUEG1's NT helix via several polar contacts[8] (Supplementary Fig. 15h). Therefore, NDUEG2 is fixated at one of the two topmost positions on Eg-CI PA, with the other one occupied by NDUEG5 (Fig. 2a). Lastly, the long CT loop of the thioredoxin-fold

subunit NDUFA2 winds among subunits NDUEG3, NDUEG1 and NDUFA6[12] (Fig. 2b and Supplementary Fig. 15i). A β-strand segment of this loop even integrates into the central β-sheet of NDUEG3's NT domain, further strengthening the association of the NDUEG3-NDUEG5 heterodimer of the FAS domain onto Eg-CI PA (Fig. 2b and Supplementary Fig. 15i).

Structural basis for Eg-CI's relative insensitivity to rotenone inhibition

Based on our structures, we propose that the much higher rotenone IC_{50} for *E. gracilis*'s CI compared to its mammalian counterparts are likely due to two residue substitutions (Supplementary Fig. 9a). Eg-CI has a Phe93-to-Pro108 substitution in NDUFS7 and a Met197-to-Lue130 substitution in NDUFS2 compared to the porcine CI[13]. $Phe93^{NDUFS7}$ is involved in interacting with the hydrophobic end of ring E in rotenone molecule bound in the deep site of Q-tunnel (ROT1 site in PDB 6ZKK, a more shallow rotenone binding site in the Q-tunnel named ROT2)[14,15]. Substituting it into a Pro with much less prominent side chain breaks the hydrophobic interaction thereby reduces the stability of rotenone binding at ROT1 site. More importantly, the NDUFS7 Met197-to-Lue130 and NDUFS2 Met197-to-Lue130 substitutions significantly constricts the diameter of the Q-tunnel just between rotenone binding sites ROT1 and ROT2. MD simulation studies have demonstrated that a kinetic barrier existed just below ROT1 site for the conformationally straight dehydrated rotenone but not for the normal rotenone[15]. Inhibition of mammalian CI by dehydrated rotenone has an IC_{50} around 570 fold higher than the normal rotenone[15]. In Eg-CI, due to the constriction of the Q-tunnel diameter, a similar kinetic barrier likely exists at approximately the same position inside the Q-tunnel for even the normal rotenone molecule. Therefore, an IC_{50} value much higher than roteonone inhibition of the mammalian CI is expected for Eg-CI. However, more precise and quantified studies have to be performed by mutagenesis and MD simulation studies.

Structural details of interactions within Eg-SC I+III_2+IV

Association interfaces between Eg-CI and Eg-$CIII_2$ are limited to two sites. Apart from NDUFA9, NDUCA1 and UQCRB, the major site also involves CI subunit NDUEG6 and $CIII_2$ subunits UQCREG1 from the proximal protomer and UQCR10 from the distal protomer (Fig. 3a). Interactions at the major CI-$CIII_2$ site feature several polar contacts contributed mostly by Eg-specific regions of the involved subunits (Supplementary Fig. 13c).

In the divergent Tt-ETC, $CIII_2$ subunit UQCRB is brought closer to NDUFA9 due to the ~28° rotation of Tt-$CIII_2$ around its C2 symmetry axis in the obligatory Tt-SC I+III_2 as compared to the mammalian counterpart[11] (Fig. 3c, and Supplementary Fig. 13a). However, the nearest distance between the two subunits is still far beyond that needed to establish any interaction (Fig. 3c). At the minor CI-$CIII_2$ site, CI subunit NDUEG9 reaches an α-helix hairpin towards the NT helix of UQCR9 (Supplementary Fig. 13d). A hydrogen bond is formed between $Arg62^{NDUEG9}$'s guanidinium group and the NT C_α-carboxyl group of UQCR9, stabilized by a cardiolipin inserted between UQCR9's NT helix and the bulk part of $CIII_2$'s membrane domain (Supplementary Fig. 13d).

The CI-CIV interaction site in the matrix features two subunits COXEG8 and COXEG9 from Eg-CIV's sepcific matrix domain, interacting with Eg-CI's NDUB9 and NDUEG8 respectively (Fig. 3a). Thereby NDUB9's role in bridging the CI-CIV association in mammalian respirasome is maintained for *E. gracilis*[16,17] (Supplementary Fig. 13f). Moreover, the Eg-specific NT loop of subunit COX4 also polarly contacts the matrix loop of NDUEG8, further reinforcing this matrix interaction site (Supplementary Fig. 13f). NDUEG7-9 line up to bridge CI-CIV interactions in the IMS (Fig. 3b,f). Among them, NDUEG7's IMS domain is structurally homologous to the permuted papain-like fold (PDB 3EBQ)[18] with density of a coordianted cation which is tentatively assigned as Zn^{2+}. The IMS domains of NDUEG7 and NDUEG8 bridge NDUB7-COX4 and ND5-COX7C associations respectively, thereby mediating the binding of SC III_2+IV onto the side of CI MA toe (Supplementary Fig. 13g,h).

The amphipathic arch at Eg-CI's MA heel

Horizontal amphipathic helices exist not only near the Q tunnel entrance but also extends to the other side of CI MA heel. Actually, amphipathic helices from NDUFS7, NDUFA12, NDUFA7, NDUEG4, NDUFS8, NDUFA3 and NDUEG10 form an amphipathic arch and create a locally deformed membrane crescent that surrounds the MA heel. Such structure is partially conserved in mammalian CI as subunits NDUFS8 and NDUFA3 also have amphipathic helices at their NTs, but lack of the Eg-specific subunit NDUEG4 and the prominent NT amphipathic helix of NDUFA7 breaks the crescent of ordered lipids[13,14]. NUDEG4 is structurally homologous to the SDR-like accessory subunit NDUFA9 but has a patch of density inside resembling mostly a lipid molecule (tentatively assigned as PC)

instead of the NADPH cofactor. If true, this lipid is pulled almost completely out of the membrane collaboratively by the hydrophobic interior of NDUEG4 and the amphipathic helix arch below, while other possibilities do exist. NUDEG4 is stabilized by subunit NDUFA7, with its NT horizontal amphipathic helix and CT helix-loop clamping it in position. NDUFA7 is a Q module accessory subunit whose most part is an extended coil[12]. In our structure, the Eg-specific CT loop of NDUFV1, NT loop and β-hairpin of NDUFS6 and NT loop of NDUEG4 not present in its homologue NDUFA9 form a loop-rich region, which helps to order the square loop of NDUFA7 thereby contributes to the stabilization of NDUEG4 indirectly.

Structural details of Eg-SC III_2+IV_2 and an alternative cyt c binding site on Eg-CIV

Apart from the universally conserved matrix subunit COX5B, the matrix domain of Eg-CIV further consists of Eg-specific matrix subunits COXEG7-10, NT extensions of Eg-specific TM subunits COXEG1 and COXEG4, as well as Eg-specific NT extensions of conserved subunits COX5C, COX7A and COX4 (Fig. 5e,f). The same space is occupied by subunits COXTT5, COXTT7 and the matrix part of COXTT1 in *T. thermophila* CIV_2, but the Eg-CIV matrix domain is more augmented and protrude deeper into the mitochondrial matrix[11]. In addition to the major Eg-$CIII_2$-CIV interaction site involving the order-disorder transition of UQCRC2's NT β-hairpin, two more interaction sites are observed in the matrix, one between $CIII_2$ subunit UQCRC1 and CIV subunits COXEG8 and COXEG9, the other between $CIII_2$ subunit UQCR10 and CIV subunits COXEG1 and COX7C (Supplementary Fig. 13j,k).

In addition to IMS loops of COX1 and the dinuclear Cu_A coordinating β-sheet cluster of COX2[19], the canonical cyt *c* binding interfaces of Eg-CIV is further flanked by a specific helmet-like domain formed mainly by the largely augmented Eg-COX6B subunit and IMS domains of COXEG3-5[20]. The Eg-specific CT loop of COX7C also extends towards the direct electron acceptor dinuclear Cu_A and participates in forming Eg-CIV's cyt *c* site (Fig. 6d,e). It is worth noting that an independent replicate Run 4 of our MD simulations also revealed other potential Eg-cyt *c* binding sites on Eg-CIV, including the concave surface of the IMS helmet domain contributed by Eg-COX6B, which could establish multiple salt bridges with the tri-helical region of Eg-cyt *c* (Supplementary Fig. 19b). However, in this binding pose, the distance between heme c of cyt *c* bound at this site and COX2's dinuclear Cu_A is far beyond the direct electron transfer distance, making this site more likely a non-

functional trap for approching cyt *c*. The simultaneous presence of functional and non-functional cytc *c* sites on Eg-CIV could represent a mechanism for specific cyt *c* recognition. Mammalian cyt *c* cannot occupy the functional cyt *c* due to potential electrostatic repulsion between its Lys26 and Eg-Lys131^{COX1} (mammalian Ala120^{COX1}), so it is more likely to be trapped on the non-functional site and unable to transfer electrons to the dinuclear Cu_A (Fig. 6d, and Supplementary Fig. 19b). In contrast, Eg-cyt *c* substitutes the lysine with valine, alleviating the electrostatic repulsion with Eg-Lys131^{COX1} and allowing binding to the functional site for electron transport to occur.

Supercomplex and possible higher order assemblies of Eg-ETC

Structural conservation of the SC III_2+IV parts in Eg-SC I+III_2+IV and Eg-SC III_2+IV_2 indicate that the assembly of Eg-SC III_2+IV likely precedes its association with Eg-CI or with another Eg-CIV copy (Supplementary Fig. 21c,d). This is unlike mammalian respirasome or SC III_2+IV, both of which assemble from individual $CIII_2$ and CIV so that CIV positions are different in respective mature supercomplexes[21]. Eg-SC III_2+IV should attach to Eg-CI after the incorporation of NDUFA9 since it provides one of the two major anchoring points for Eg-SC III_2+IV (Supplementary Fig. 21b,d). For mammalian respirasome, mere P_D-a module, providing two CI-$CIII_2$ interaction sites in both the matrix and IMS, is sufficient to promote $CIII_2$ and CIV interactions[17,22]. However, conformational changes and/or participation of assembly factors not present in the mature *E. gracilis* supercomplexes cannot be neglected, making the real assembly procedure more complicated than described here. Interestingly, existance of Eg-SC I+III_2+IV_2 has been previously suggested[23], meanwhile structural alignment of Eg-SC I+III_2+IV and Eg-SC III_2+IV_2 by the SC III_2+IV part identified no obvious steric clashes (Supplementary Fig. 21c). Although a minor particle set correponding to Eg-SC (III_2+IV_2)$_2$ is clearly visible in our 2D classes, no Eg-SC I+III_2+IV_2 particle seem to exist (Supplementary Figs. 2 and 3). The C2 symmetry of Eg-SC III_2+IV_2 would further theoretically allow assoication of another copy of Eg-CI onto its unoccupied surface, creating an Eg-MC I_2+III_2+IV_2 similar to the *T. thermophila* MC IV_2+(I+III_2+II)$_2$[24]. Lack of biochemical evidence and the existance of Eg-SC (III_2+IV_2)$_2$ argue against such megacomplex, whose apparent non-existance or extreme low adundance can be due to the dissociative effect of digionin, or can be explained by the unique euglenozoan ETC assembly pathway in adaptation to the discoidal cristae, as has been demonstrated vividly for the ciliate ETC[24–27].

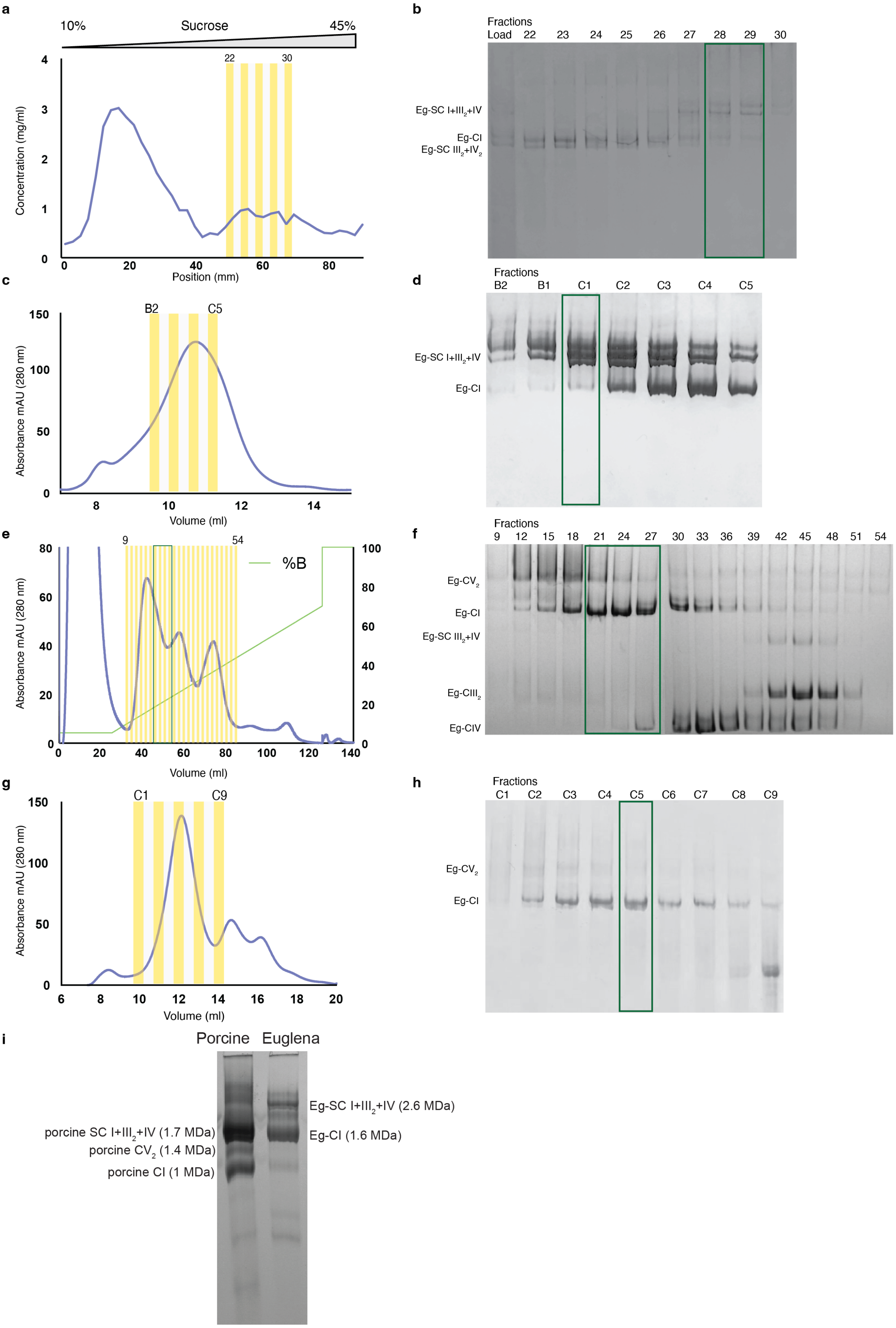

Supplementary figure 1. Purification of *E. gracilis*'s ETC supercomplexes and complex I. a,b, Sucrose gradient ultracentrifugation fractionation (**a**) and corresponding BN-PAGE (**b**) of digitonin-

extracted *E. gracilis* supercomplexes. Fractions pooled and loaded onto SEC chromatography are highlighted by green boxes. **c,d,** Superose 6 Increase 10/300 GL column chromatograms (**c**) and corresponding BN-PAGE (**d**) of pooled fractions from ultracentrifugation. **e,f,** Ion exchange chromatography (**e**) and corresponding BN-PAGE (**f**) of LMNG-extracted Eg-CI. Fractions pooled and loaded onto SEC chromatography are highlighted by green boxes. **g,h,** Superose 6 Increase 10/300 GL column chromatograms (**g**) and corresponding BN-PAGE (**h**) of pooled fractions from ion exchange chromatography. **i**, Comparison of molecular weights between *Sus scrofa*'s and *E. gracilis*'s ETC complexes. Fractions used for cryo-EM grid preparations are highlighted by green boxes. All BN-PAGE gels shown here are stained by complex I activity. Source data are provided as a Source Data file.

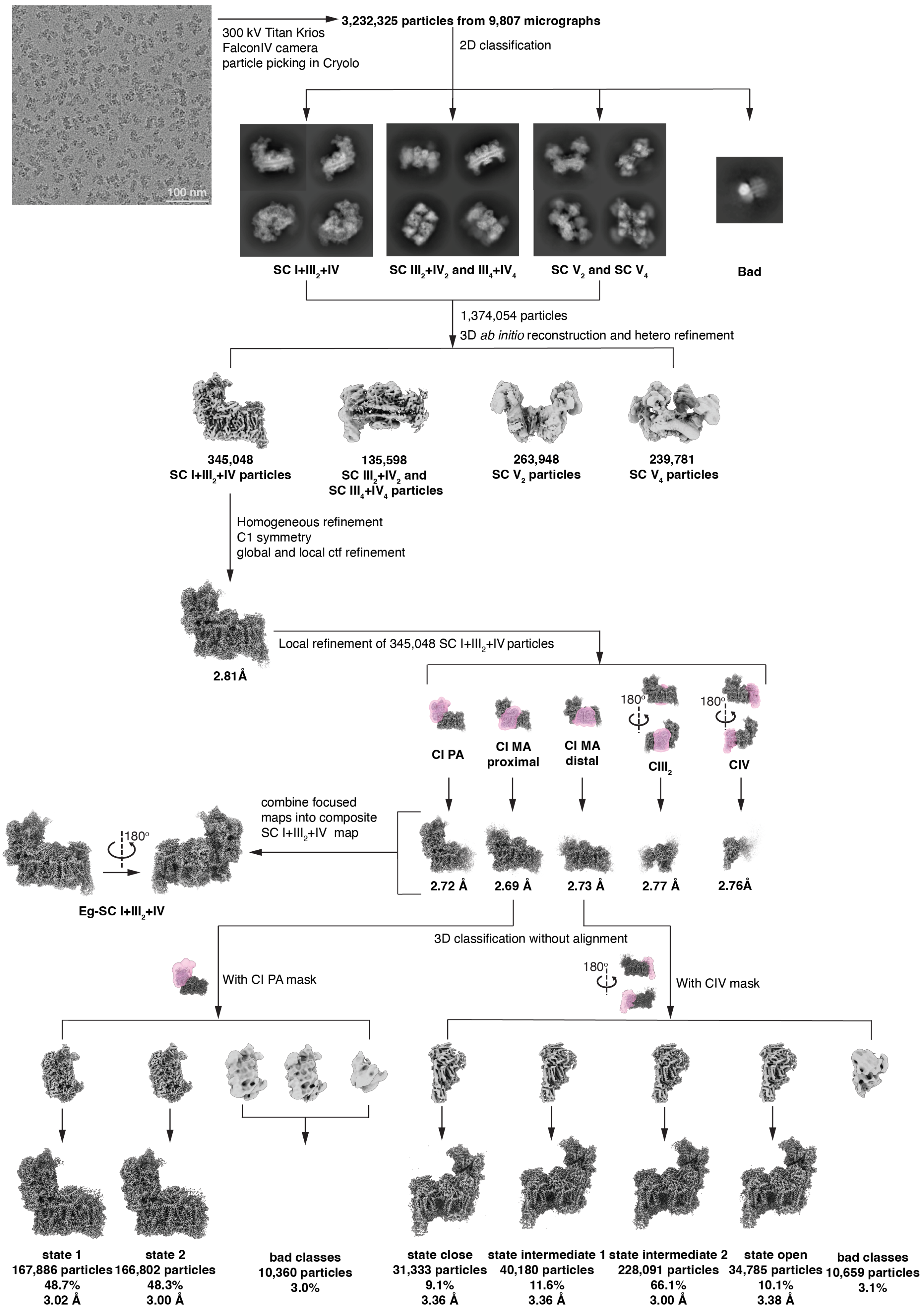

Supplementary figure 2. Cryo-EM image processing of Eg-SC I+III_2+IV. A total of 9,807 images were collected from a 300 kV Titan Krios microscope equipped with Falcon IV camera, from which 3,232,325 particles were initially picked using crYOLO. In cryoSPARC, a total of 345,048 Eg-SC I+III_2+IV particles were selected after 2D classification, from which 345,048 Eg-SC I+III_2+IV particles were obtained after

heterogeneous refinement. Overall homogenous refinement gave a resolution of 2.81 Å. Local refinements of Eg-CI PA, proximal and distal regions of CI MA, $CIII_2$ and CIV regions gave resolutions from 2.69-2.76 Å, from which a composite Eg-I+III_2+IV map was generated in Phenix. 3D classification without alignment using CI PA mask gave two good classes noted as state 1 and state 2, and refined to overall resolutions of 3.02 Å and 3.00 Å, respectively. 3D classification without alignment using Eg-CIV mask gave four good classes with increasing $CIII_2$-CIV angles which were noted as close class, intermediate 1 class, intermediate 2 class and open class, and refined to an overall resolution of 3.36 Å, 3.36 Å, 3.00 Å and 3.38 Å, respectively. Masks used for individual local refinements are indicated as pink transparent surfaces.

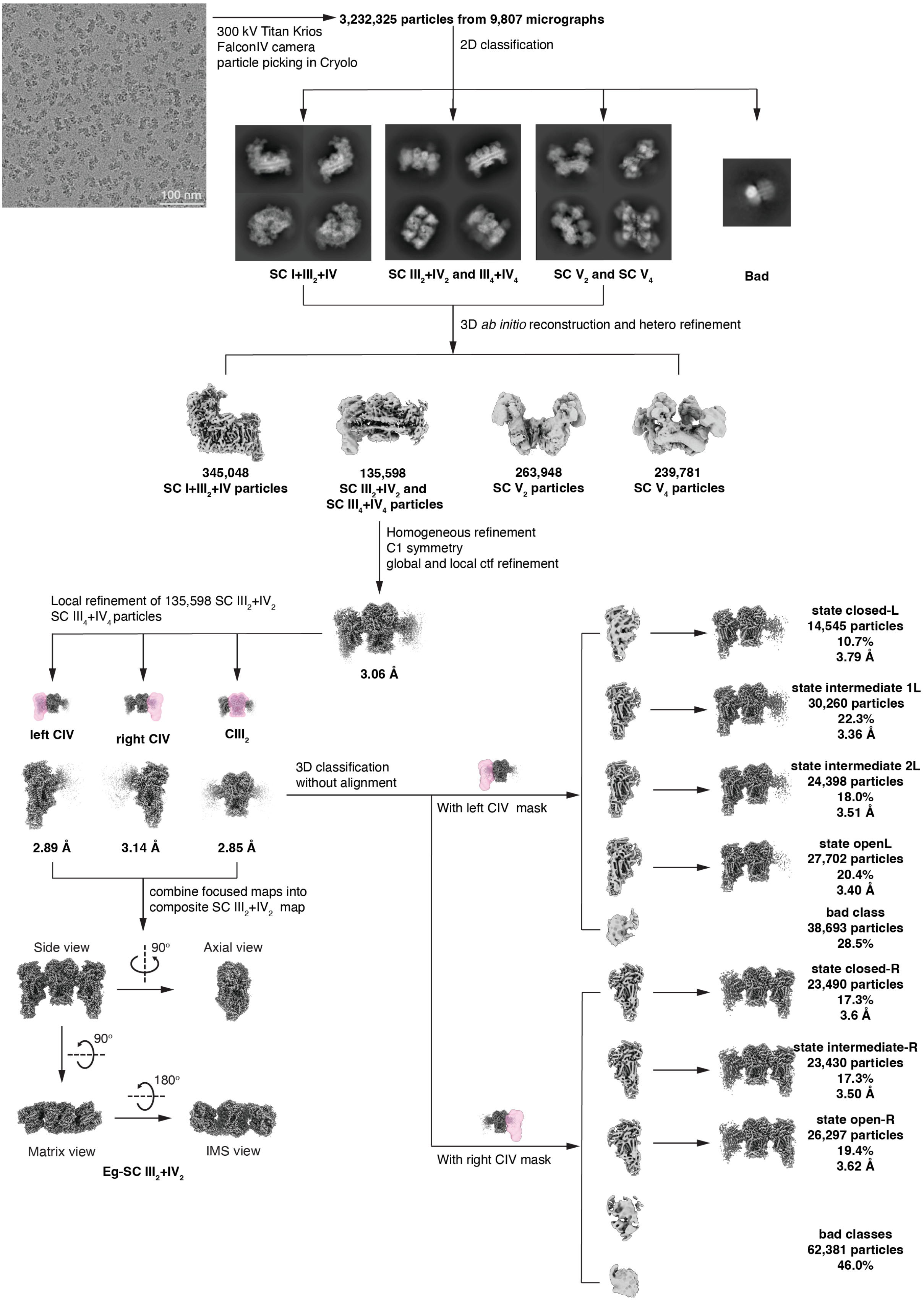

Supplementary figure 3. Cryo-EM image processing of Eg-SC III_2+IV_2. Processing steps before 3D refinements were the same as **Supplementary** Fig. 4. In cryoSPARC, a total of 135,598 Eg-SC III_2+IV_2 and Eg-SC III_4+IV_4 particles were obtained after heterogeneous refinement and refined to an overall resolution of 3.06 Å using a SC III_2+IV_2 mask. Local refinements of $CIII_2$ and the two CIVs gave

resolutions from 2.85-3.14 Å, from which a composite Eg-III_2+IV_2 map was generated in Phenix. The 3D classifications without alignment using the left and right CIV masks gave four and three classes respectively. They were noted as indicated in the figure. Masks used for individual local refinements are indicated as pink transparent surfaces.

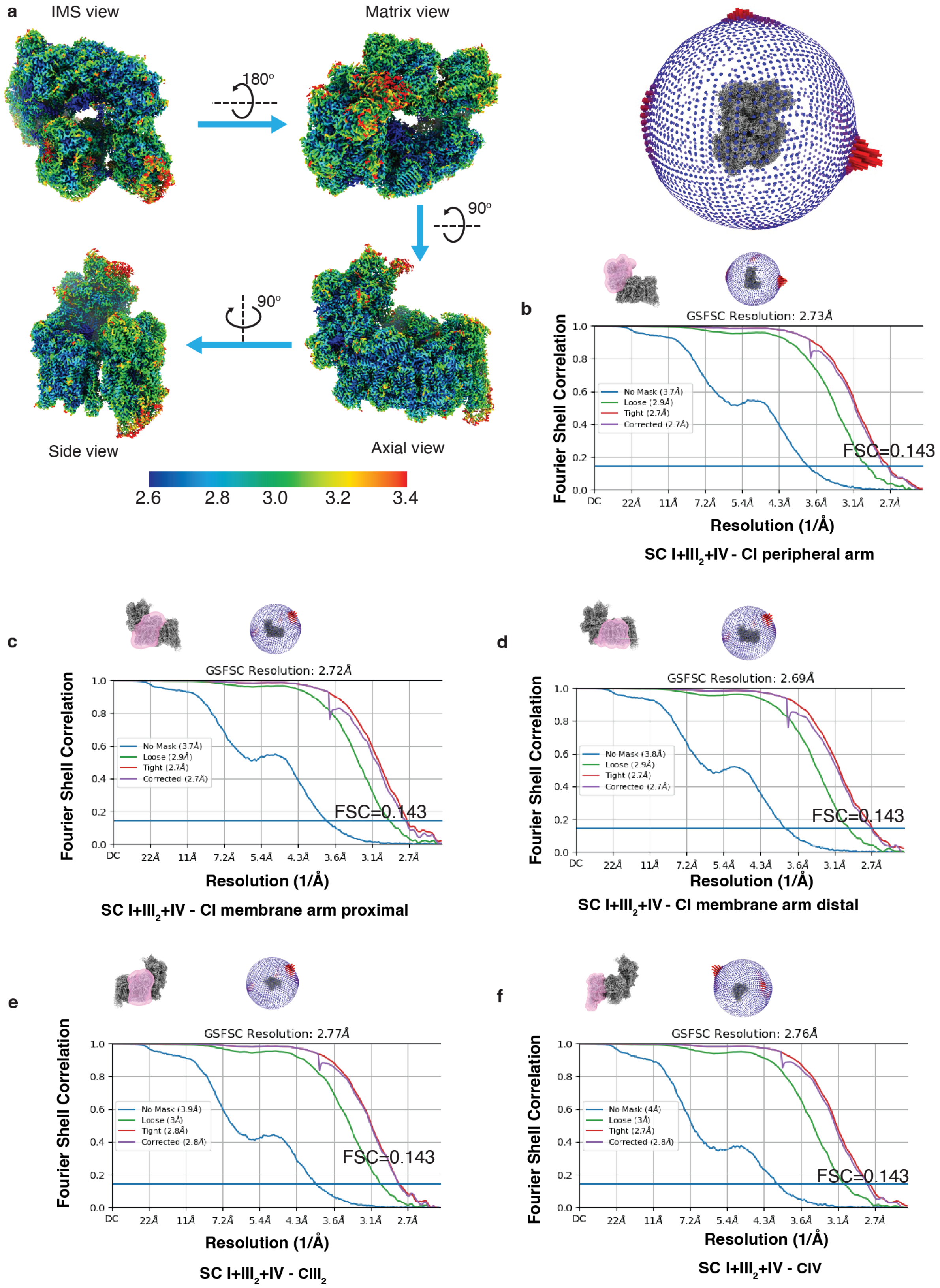

Supplementary figure 4. Local resolution maps and Fourier shell correlation (FSC) **curves of Eg-SC I+III$_2$+IV. a**, Local resolution plotted on Eg-SC I+III$_2$+IV composite map at different views. **b-f**, FSC curves (gold standard FSC=0.143 for resolution estimation) of different local refinements of Eg-SC I+III$_2$+IV.

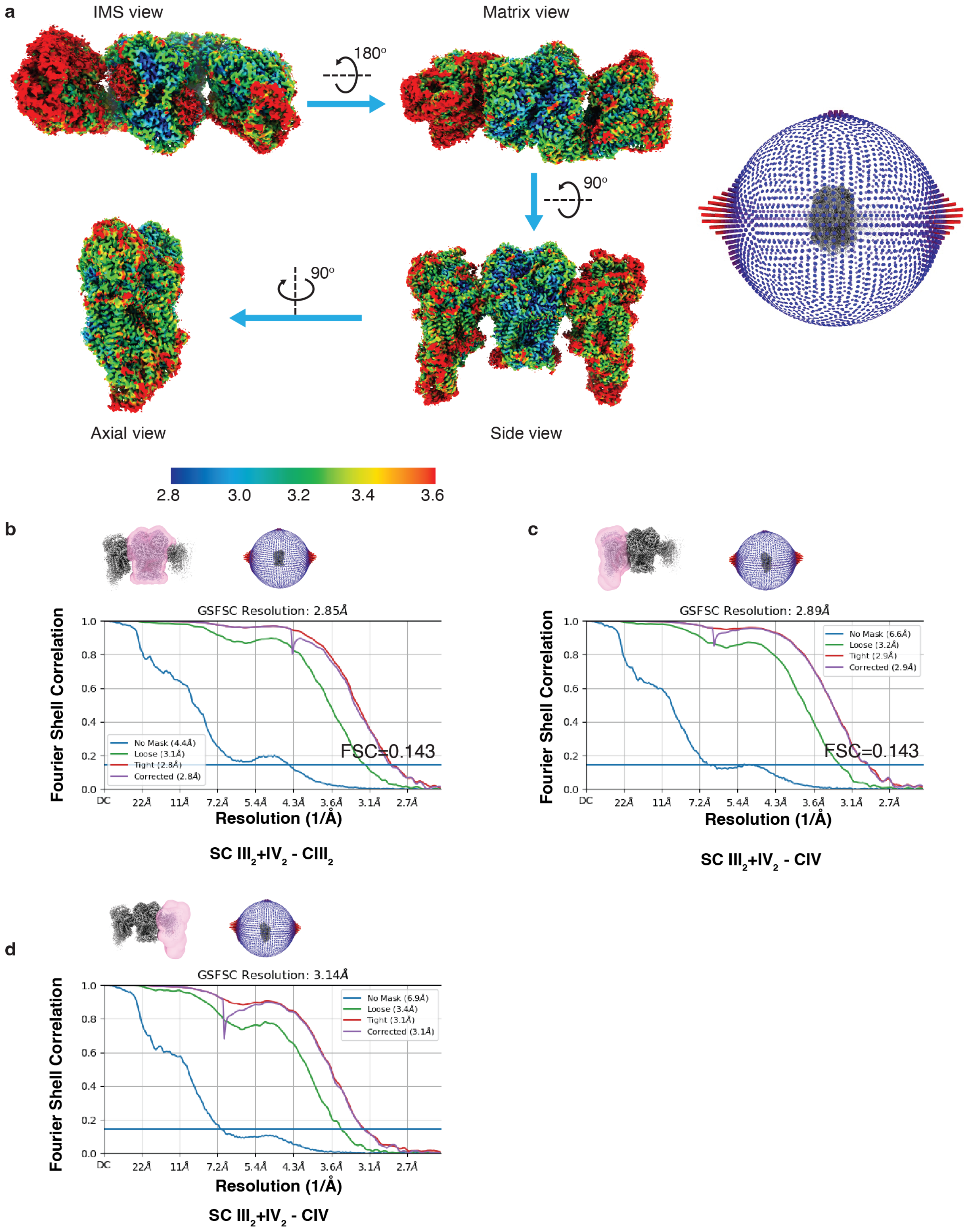

Supplementary figure 5. Local resolution maps and Fourier shell correlation (FSC) curves of Eg-SC III_2+IV_2. a, Local resolution plotted on Eg-SC III_2+IV_2 composite maps at different views. **b-d**, FSC curves (gold standard FSC=0.143 for resolution estimation) of different local refinements of Eg-SC III_2+IV_2.

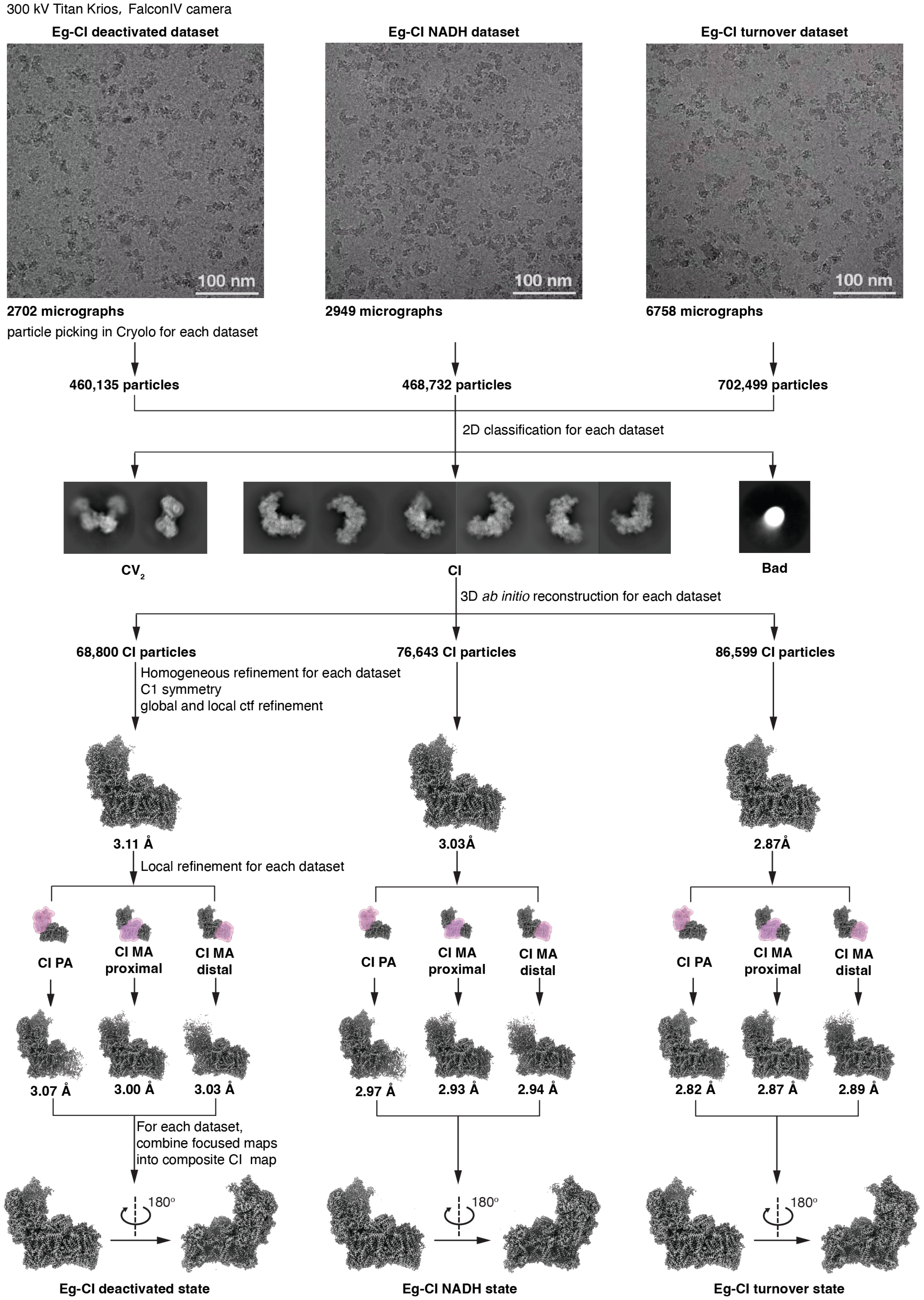

Supplementary figure 6. Cryo-EM image processing of Eg-CI in deactive, NADH-reduced and turnover states. A total of 2,702, 2,949 and 6,758 images were collected from a 300 kV Titan Krios microscope equipped with Falcon IV camera, from which 460,135, 468,732 and 702,499 CI particles were initially picked using crYOLO for Eg-deactive, NADH and turnover datasets, respectively. In cryoSPARC, a total of 68,800, 76,643 and 86,599 CI particles were obtained after 2D classification and refined to

overall resolutions of 3.11 Å, 3.03 Å and 2.87 Å, respectively. Local refinements of CI PA and proximal and distal regions of Eg-CI MA gave resolutions from 2.82-3.07 Å, from which composite Eg-CI maps were generated in Phenix for each state. Masks used for individual local refinements are indicated as pink transparent surfaces.

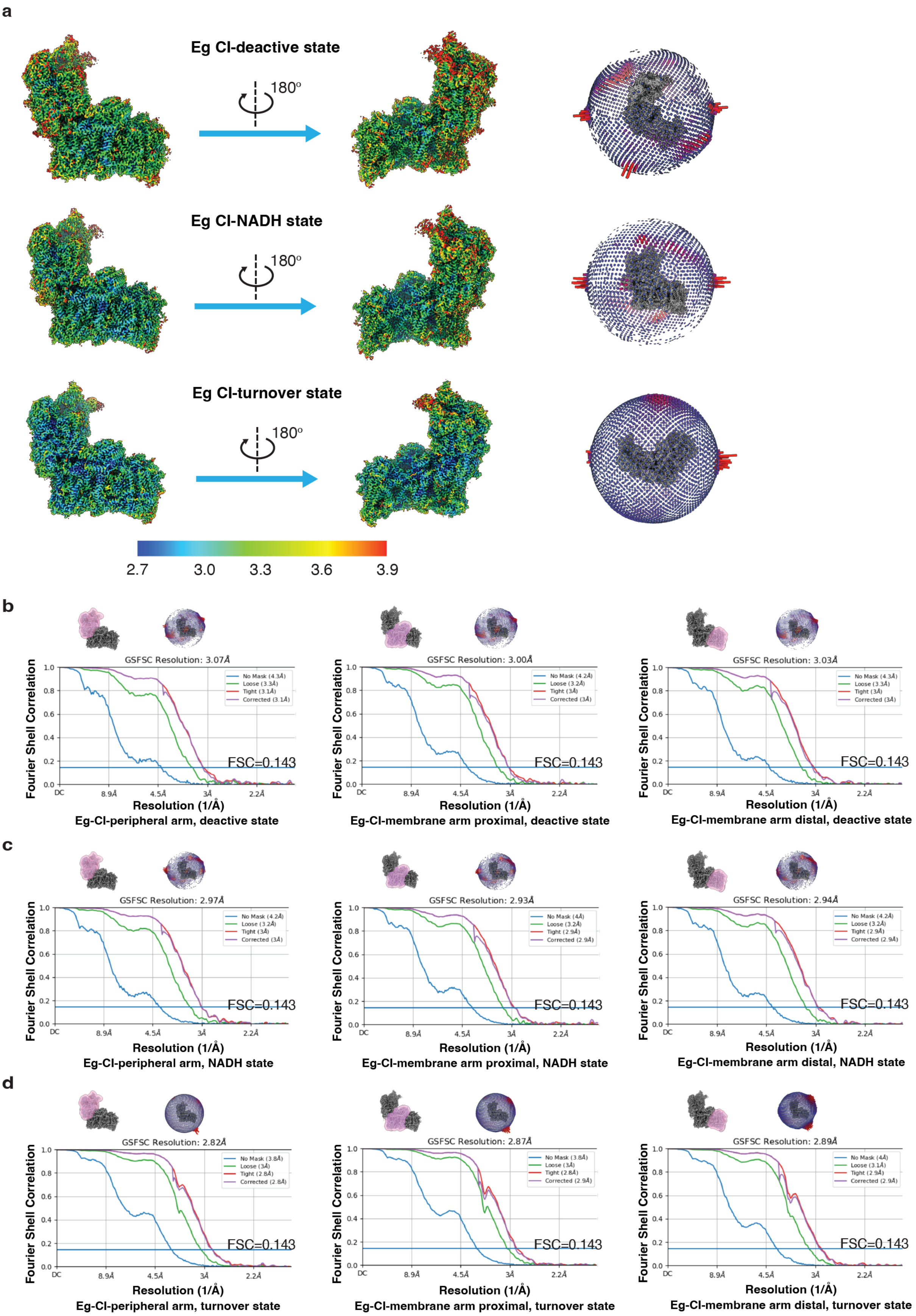
a
Eg CI-deactive state
180°
Eg CI-NADH state
180°
Eg CI-turnover state
180°
2.7
3.0
3.3
3.6
3.9
b
GSFSC Resolution: 3.07Å
No Mask (4.3Å)
Loose (3.3Å)
Tight (3.1Å)
Corrected (3.1Å)
FSC=0.143
Fourier Shell Correlation
Resolution (1/Å)
Eg-CI-peripheral arm, deactive state
GSFSC Resolution: 3.00Å
No Mask (4.2Å)
Loose (3.2Å)
Tight (3Å)
Corrected (3Å)
FSC=0.143
Fourier Shell Correlation
Resolution (1/Å)
Eg-CI-membrane arm proximal, deactive state
GSFSC Resolution: 3.03Å
No Mask (4.3Å)
Loose (3.3Å)
Tight (3Å)
Corrected (3Å)
FSC=0.143
Fourier Shell Correlation
Resolution (1/Å)
Eg-CI-membrane arm distal, deactive state
c
GSFSC Resolution: 2.97Å
No Mask (4.2Å)
Loose (3.2Å)
Tight (3Å)
Corrected (3Å)
FSC=0.143
Fourier Shell Correlation
Resolution (1/Å)
Eg-CI-peripheral arm, NADH state
GSFSC Resolution: 2.93Å
No Mask (4Å)
Loose (3.2Å)
Tight (2.9Å)
Corrected (2.9Å)
FSC=0.143
Fourier Shell Correlation
Resolution (1/Å)
Eg-CI-membrane arm proximal, NADH state
GSFSC Resolution: 2.94Å
No Mask (4.2Å)
Loose (3.2Å)
Tight (2.9Å)
Corrected (2.9Å)
FSC=0.143
Fourier Shell Correlation
Resolution (1/Å)
Eg-CI-membrane arm distal, NADH state
d
GSFSC Resolution: 2.82Å
No Mask (3.8Å)
Loose (3Å)
Tight (2.8Å)
Corrected (2.8Å)
FSC=0.143
Fourier Shell Correlation
Resolution (1/Å)
Eg-CI-peripheral arm, turnover state
GSFSC Resolution: 2.87Å
No Mask (3.8Å)
Loose (3Å)
Tight (2.8Å)
Corrected (2.9Å)
FSC=0.143
Fourier Shell Correlation
Resolution (1/Å)
Eg-CI-membrane arm proximal, turnover state
GSFSC Resolution: 2.89Å
No Mask (4Å)
Loose (3.1Å)
Tight (2.9Å)
Corrected (2.9Å)
FSC=0.143
Fourier Shell Correlation
Resolution (1/Å)
Eg-CI-membrane arm distal, turnover state

Supplementary figure 7. Local resolution maps and Fourier shell correlation (FSC) curves of Eg-CI in deactive, NADH-reduced and turnover states. a, Local resolution plotted on Eg-CI composite maps under deactive, NADH-reduced and turnover conditions, at different views. **b-d**, FSC curves (gold standard FSC=0.143 for resolution estimation) of different local refinements of Eg-CI in deactive (**b**), NADH-reduced (**c**) and turnover (**d**) states.

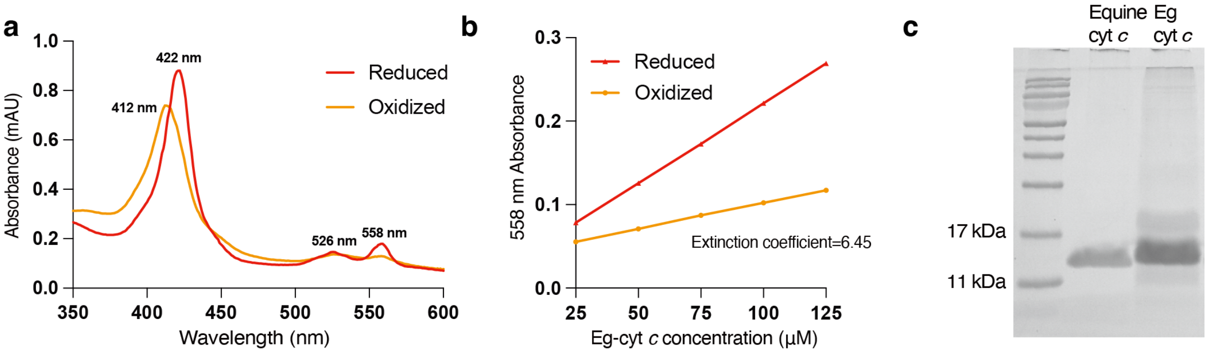

Supplementary figure 8. Recombinant expression and extinction coefficient measurement of Eg-cyt *c*. **a**, 350-600 nm spectral scan of recombinantly expressed Eg-cyt *c* shows characteristic shift of Soret peak (412 nm for oxidized cyt *c*, 422 nm for reduced cyt *c*) and changes in the Q-band region (526-558 nm). The cyt *c* was reduced and oxidized by DDT and H_2O_2 (v/v) respectively. **b**, Standard curves for reduced and oxidized Eg-cyt *c* at increasing concentrations monitored at 558 nm. Data are presented as mean values ± standard error of mean (SEM), n=3 biologically independent activity experiments. Note that error bars are present but too small to be discerned here. **c**, SDS-PAGE of equine cyt *c* and recombinantly expressed Eg-cyt *c*, stained by Coomassie brilliant blue. Source data are provided as a Source Data file.

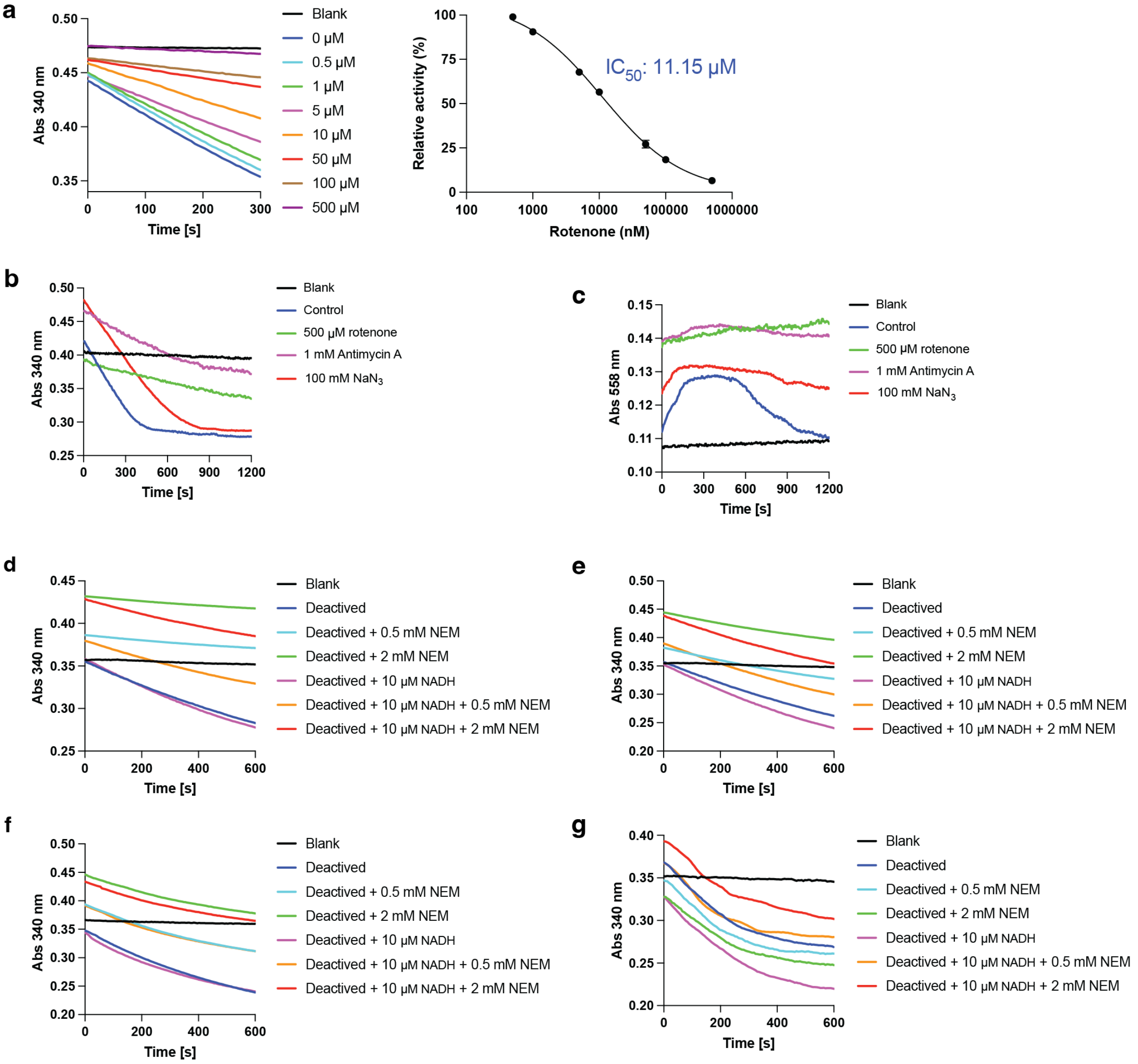

Supplementary figure 9. Kinetic curves for activity and A/D transition assays of Eg-supercomplex and Eg-CI. a, Kinetic curves (left) and dose response curves (right) for rotenone inhibition of Eg-CI activity monitored by NADH oxidation at 340 nm. Regression to the standard dose response inhibition curve to generate the IC_{50} value was calculated using GraphPad Prism version 8.0.0 (n=3). **b**,**c**, Kinetic curves for Eg-supercomplex activity measurements in **Fig. 1f-h**. Note the NADH oxidation rates monitored at 340 nm (**b**) and cyt *c* reduction rates monitored at 558 nm (**c**) are calculated by slopes of the initial linear phases in respective kinetic curves. Cyt *c* oxidation rates monitored at 558 nm (**c**) are calculated by slopes of linear regions in the last decline phase in respective kinetic curves as described in the **Materials and Methods** section. **d-g**, Kinetic curves for A/D transition assays in **Fig. 4a-d**. NADH oxidation activities were monitored at 340 nm for thermally deactivated *S. scrofa* CI (**d**), *S. scrofa* SC $I+III_2+IV$ (**e**), Eg-CI (**f**) and Eg-SC $I+III_2+IV$ (**g**) in the presence of 0, 0.5 or 2 μM NEM, without or with re-activation by 10 μM NADH. For (**a-g**), data are presented as mean values ± standard error of mean (SEM), n=3 biologically independent activity experiments, error bars representing SEM are not shown for clarity. Source data are provided as a Source Data file.

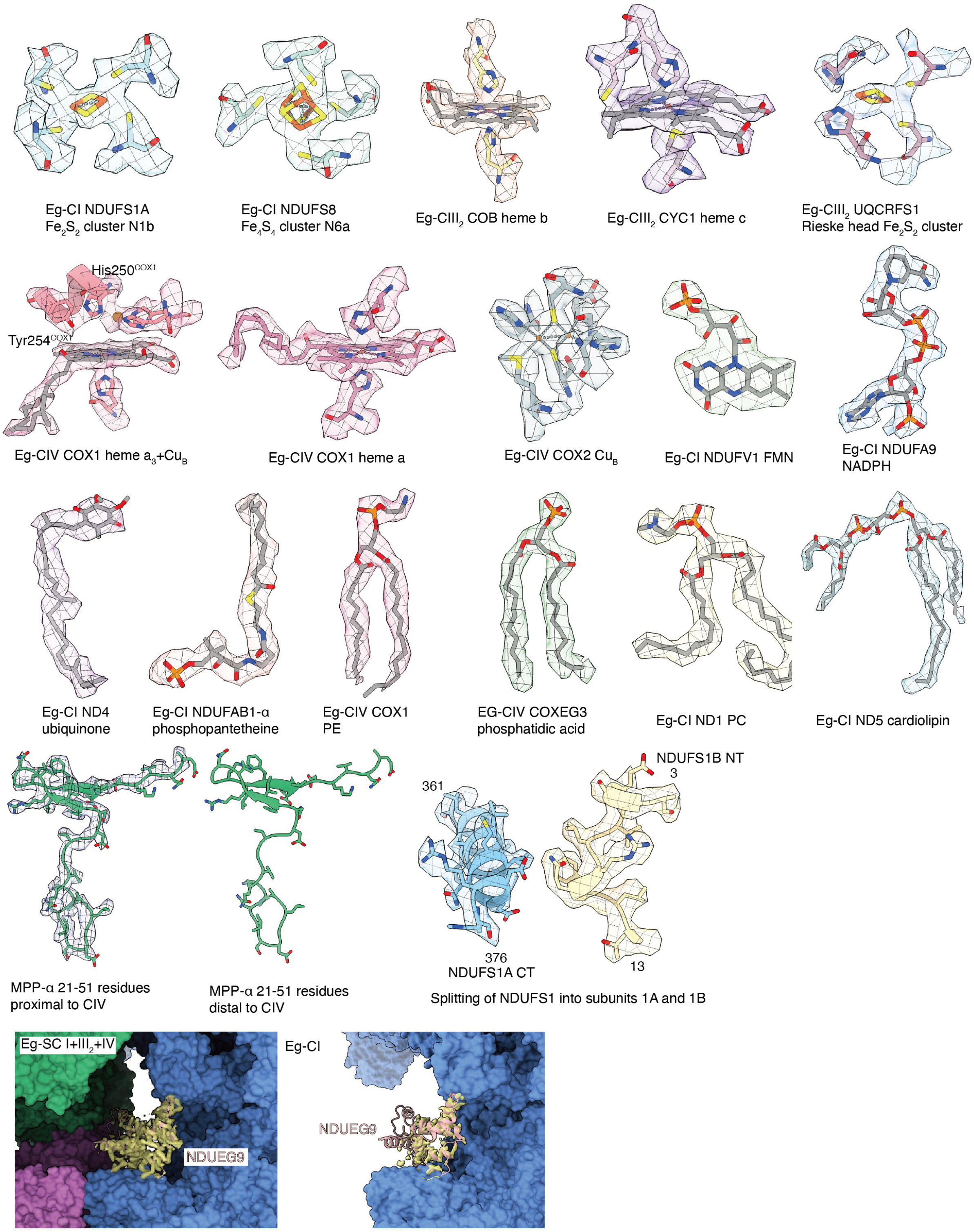

Supplementary figure 10. Representative Cryo-EM densities of Eg-SC I+III_2+IV. Representative densities were shown as transparent surfaces layered by black meshes, for different co-factors and lipids. Densities were also shown for regions corresponding to residues 21-51 in the proximal and distal MPP-α subunits of Eg-SC I+III_2+IV. Densities for the NDUFS1A CT and NDUFS1B NT regions were also shown to illustrate genuine splitting of core subunit NDUFS1 into two subunits. Densities for subunit NDUEG9 in Eg-SC I+III_2+IV and Eg-CI are colored yellow in solid surface background for clarity.

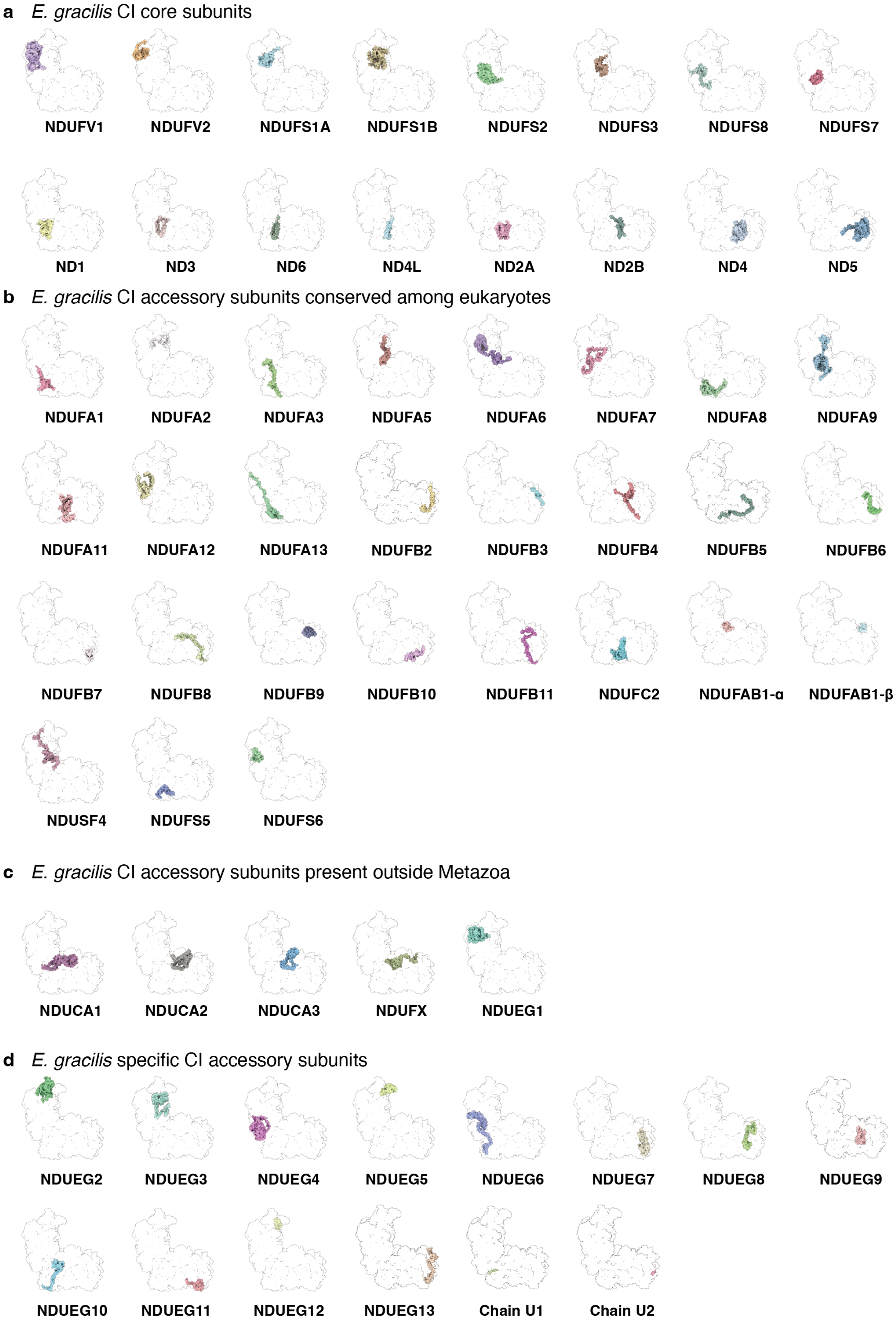

Supplementary figure 11. Individual subunits of Eg-CI. a-d, Core (**a**), universally conserved (**b**), non-Metazoan conserved (**c**) and Eg-specific (**d**) subunits of Eg-CI are shown in colored surfaces as in **Fig. 1**, with the whole complex shown in silhouettes.

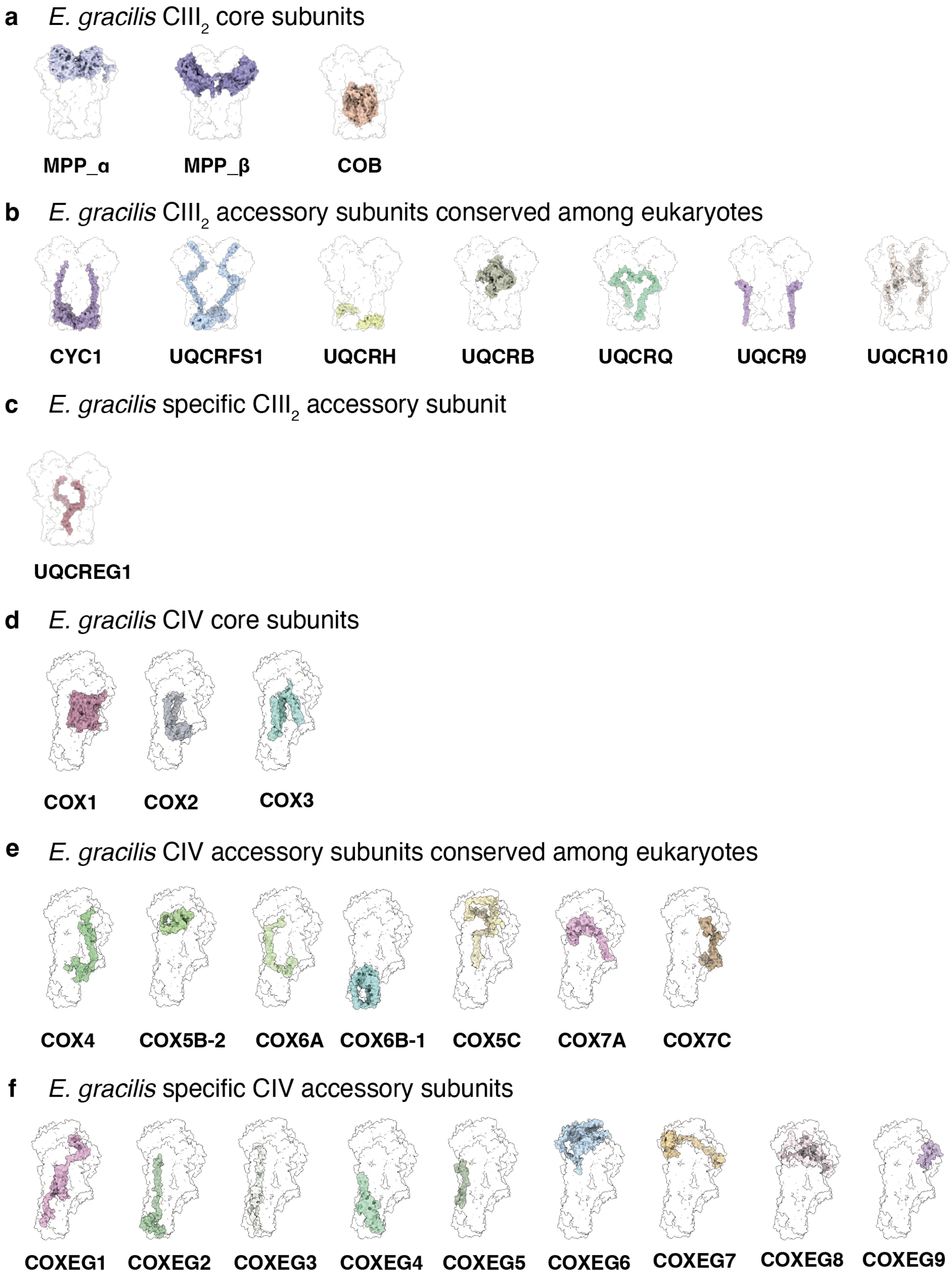

Supplementary figure 12. Individual subunits of Eg-CIII$_2$ and Eg-CIV. a-f, Eg-CIII$_2$ core (**a**), conserved (**b**), Eg-specific (**c**) subunits and Eg-CIV core (**d**), conserved (**e**), Eg-specific (**f**) subunits are shown in colored surface as in **Fig. 1**, with the whole complexes shown in silhouettes.

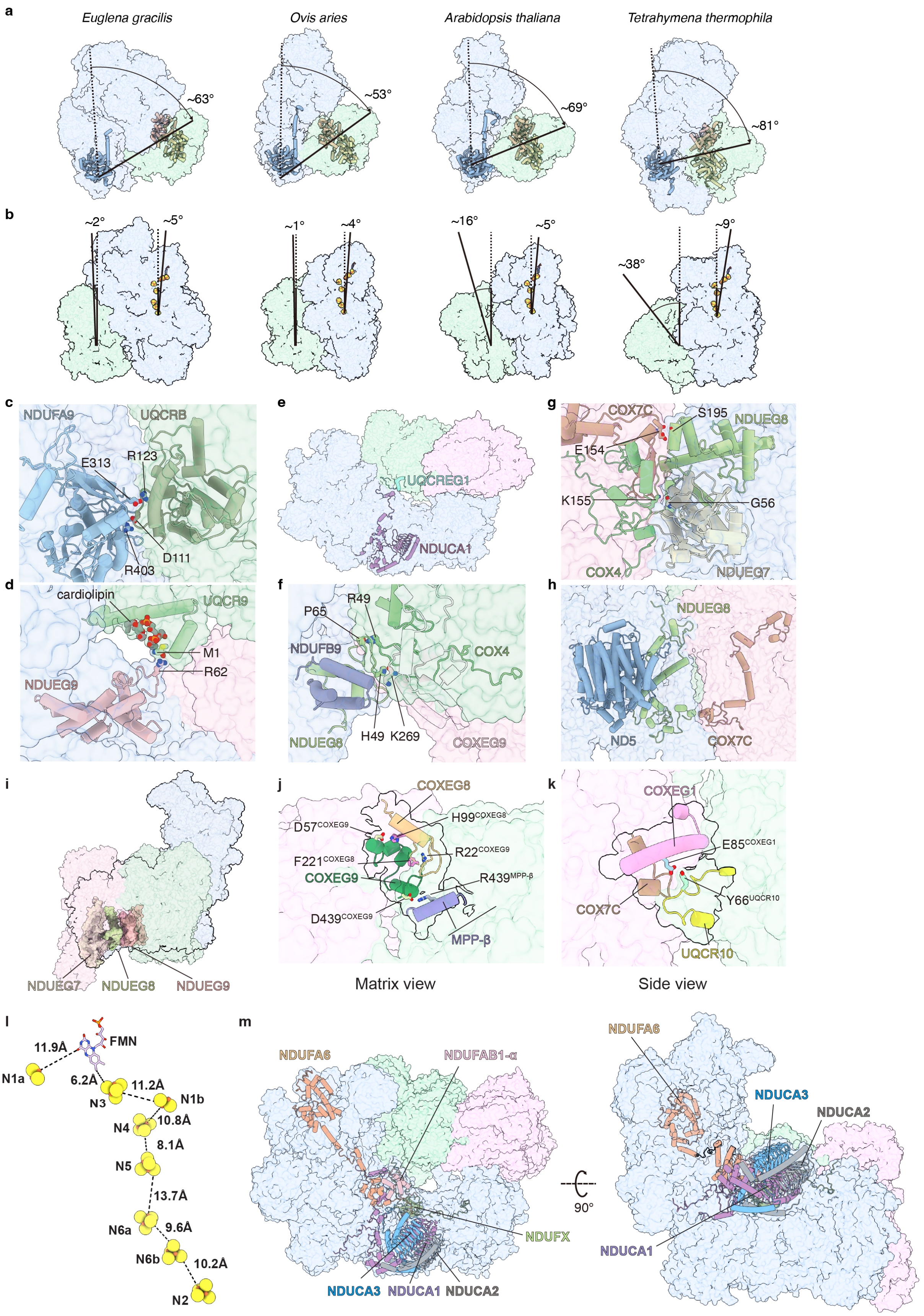

Supplementary figure 13. Architectures of different SC I+III$_2$ and interaction sites within Eg-SC I+III$_2$+IV and III$_2$+IV$_2$. a, Comparison of CI-CIII$_2$ angles of SC I+III$_2$ from different species viewed from the matrix. The dotted lines indicate the approximate long axis of the CI MA; the solid lines are the transverse axes between two COB subunits of CIII$_2$. **b**, Angles of the vertical axes of CI PA and CIII$_2$

(solid lines) relative to the membrane normal (dotted lines) of SC I+III_2 from different species viewed alongside the membrane. The structures of the *O. aries*, *A. thaliana* and *T. thermophile* SC I+III_2 are from PDB 6QBX, 8BPX and 7TGH. **c-h**, Zoom-ins of the CI-$CIII_2$ major site (**c** and **e**), CI-$CIII_2$ minor site (**d**), CI-CIV matrix site (**f**) and CI-CIV IMS sites (**g** and **h**). Key subunits are shown as cartoons and colored individually. Key residues forming polar contacts are shown as sticks and colored by elements. **i**, Positions of the NDUEG7, NDUEG8, NDUEG9 and NDUFA9, shown in solid surfaces, in Eg-SC I+III_2+IV. **j**,**k**, Zoom-ins of Eg-$CIII_2$+CIV_2 interaction sites 2 (**j**), and 3 (**k**). Key residues forming polar contacts are shown as sticks and colored by elements. **l**, The electron transfer path of *E. gracilis* CI. The FeS clusters of *E. gracilis* are shown as atoms colored by the element. **m**, Positions of the γ-carbonic anhydrase domain and the ferredoxin bridge domain of *E. gracilis* CI in Eg-SC I+III_2+IV. Key subunits are shown as cartoons and colored individually.

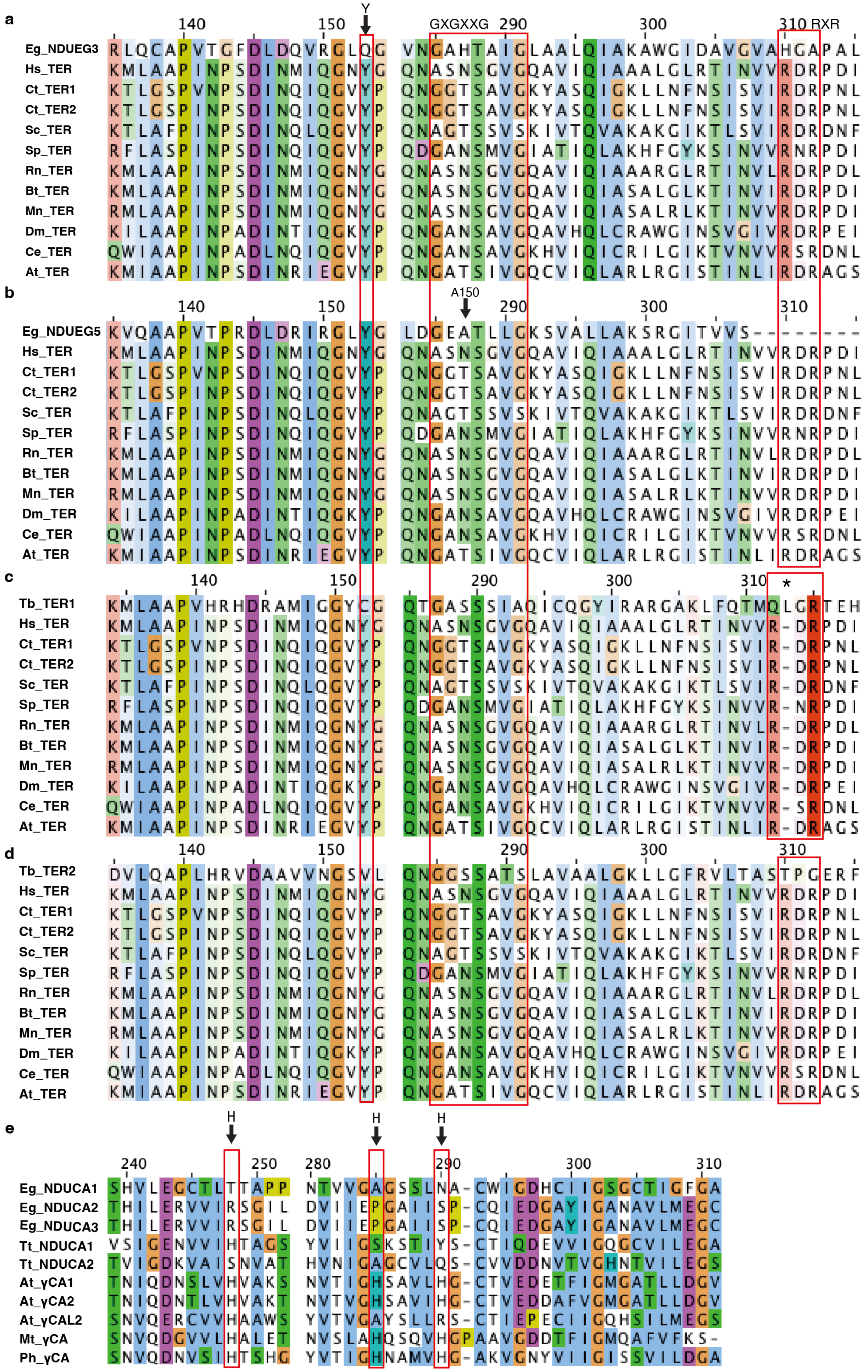
a
140
150
Y
GXGXXG
290
300
310 RXR
Eg_NDUEG3
Hs_TER
Ct_TER1
Ct_TER2
Sc_TER
Sp_TER
Rn_TER
Bt_TER
Mn_TER
Dm_TER
Ce_TER
At_TER
b
140
150
A150
290
300
310
Eg_NDUEG5
Hs_TER
Ct_TER1
Ct_TER2
Sc_TER
Sp_TER
Rn_TER
Bt_TER
Mn_TER
Dm_TER
Ce_TER
At_TER
c
140
150
290
300
310
*
Tb_TER1
Hs_TER
Ct_TER1
Ct_TER2
Sc_TER
Sp_TER
Rn_TER
Bt_TER
Mn_TER
Dm_TER
Ce_TER
At_TER
d
140
150
290
300
310
Tb_TER2
Hs_TER
Ct_TER1
Ct_TER2
Sc_TER
Sp_TER
Rn_TER
Bt_TER
Mn_TER
Dm_TER
Ce_TER
At_TER
e
H
H
H
240
250
280
290
300
310
Eg_NDUCA1
Eg_NDUCA2
Eg_NDUCA3
Tt_NDUCA1
Tt_NDUCA2
At_γCA1
At_γCA2
At_γCAL2
Mt_γCA
Ph_γCA

Supplementary figure 14. Sequence alignments of MDR-like FAS subunits and γ-carbonic anhydrase subunits among species. a-d, Sequence alignments of MDR-like FAS subunits f *E. gracilis* CI and *T. brucei* CI to known TERs. Sequences of Eg-CI NDUEG3 (**a**), Eg-CI NDUEG5 (**b**) and the two MDR-like subunits from Tb-CI (**c** and **d**) are aligned to known TER sequences. Positions for the critical Y55, NADH-contacting GXGXXG motif and NADPH-contacting RXR motif are marked by red boxes. TER sequences used include *H. sapiens* (Q9BV79), *C. tropicalis* (Q8WZM3 and Q8WZM4), *S. cerevisiae* (P38071), *S. pombe* (Q10488), *R. norvegicus* (Q9Z311), *B. taurus* (XP_024855125), *M. musculus* (XP_006538948), *D. melanogaster* (Q9V6U9), *C. elegans* (O45903), and *A. thaliana* (Q8LCU7). **e,** Sequence alignments of γ-carbonic anhydrase from *E. gracilis*, *T. thermophila*, *A. thaliana*, *M. thermophila* and *P. horikoshii*. Positions for the critical histidine are marked by red boxes. γ-CA sequences used include *T. thermophila* (Q22XU5 and I7M6S0), *A. thaliana* (Q9FWR5, Q9C6B3 and Q9SMN1), *M. thermophila* (P40881) and *P. horikoshii* (O59257).

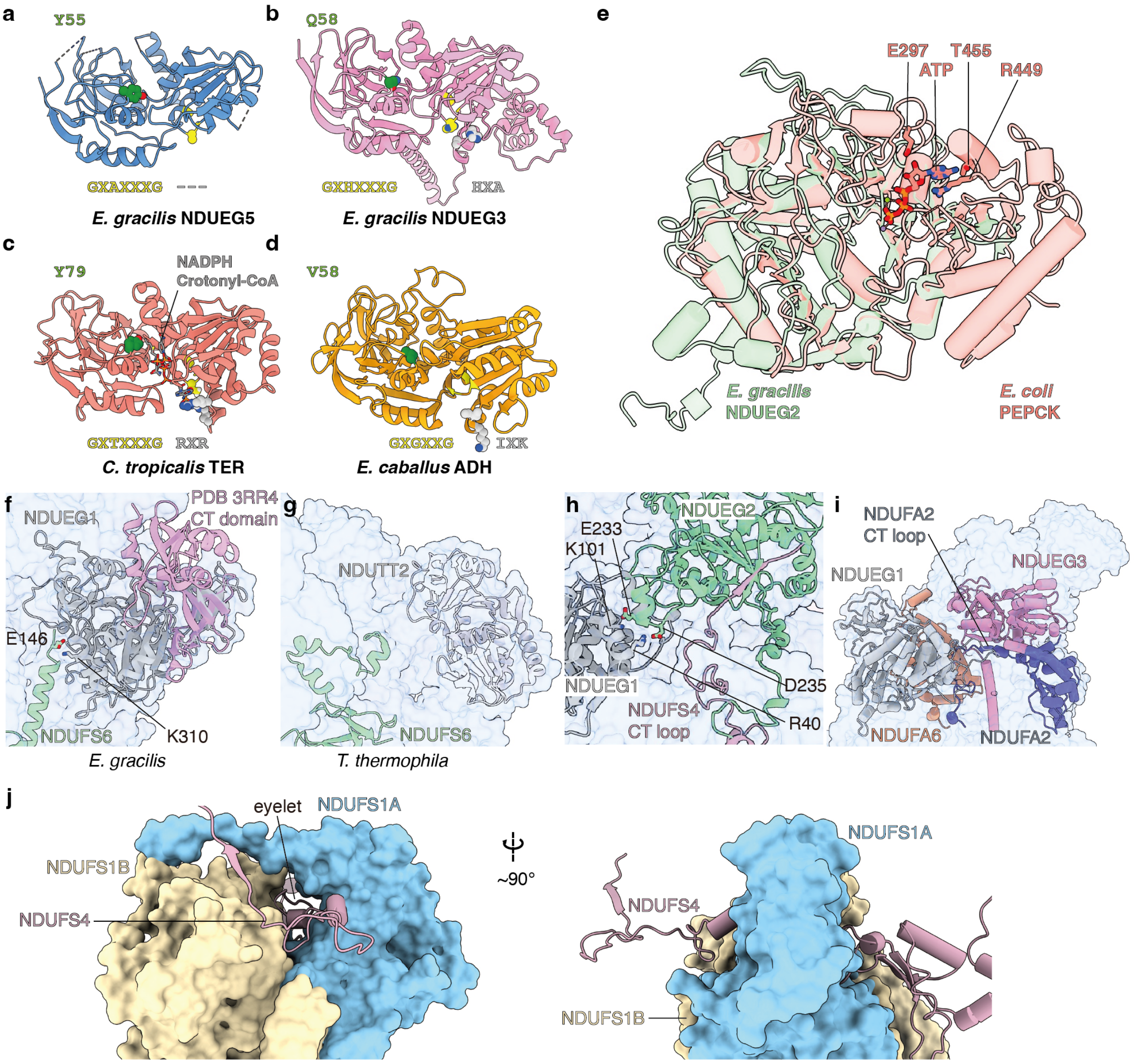

Supplementary figure 15. The FAS domain of *E. gracilis* CI. a-**d**, Comparison of Eg-NDUEG3 (**a**) and Eg-NDUEG5 (**b**) to structures of *C. tropicalis* TER (PDB 4WAS) (**c**) and *E. caballus* ADH (PDB 1HET) (**d**). Key residues and NADPH are shown as atomic spheres and sticks respectively and colored by elements. **e**, Alignment of Eg-NDUEG2 and the structure of *E. coli* PEPCK (PDB 1AQ2). Key residues and ATP are shown as atomic sticks and colored by elements. **f**, The CT domain of mycobacterial AMP-forming acyl-CoA synthetase (PDB 3R44) is aligned to Eg-NDUEG1, while its NT domain is hidden for clarity. Note that NDUEG1 interacts with NDUFS6 in Eg-CI. **g**, The interaction between NDUFS6 and NDUTT2, structural homolog of Eg-NDUEG1, is not present in *T. thermophila* CI (PDB 7TGH). **h**, A double helix bundle formed by NDUEG1 and NDUEG2. Key interacting residues are shown as stick and colored by elements. **i**, The NDUFA2 CT loop integrates into the central β-sheet of NDUEG3's NT domain. **j**, NDUFS4's CT loop, shown as cartoon in salmon, threads through an eyelet formed by NDUFS1A and NDUFS1B, shown as blue and yellow surfaces respectively.

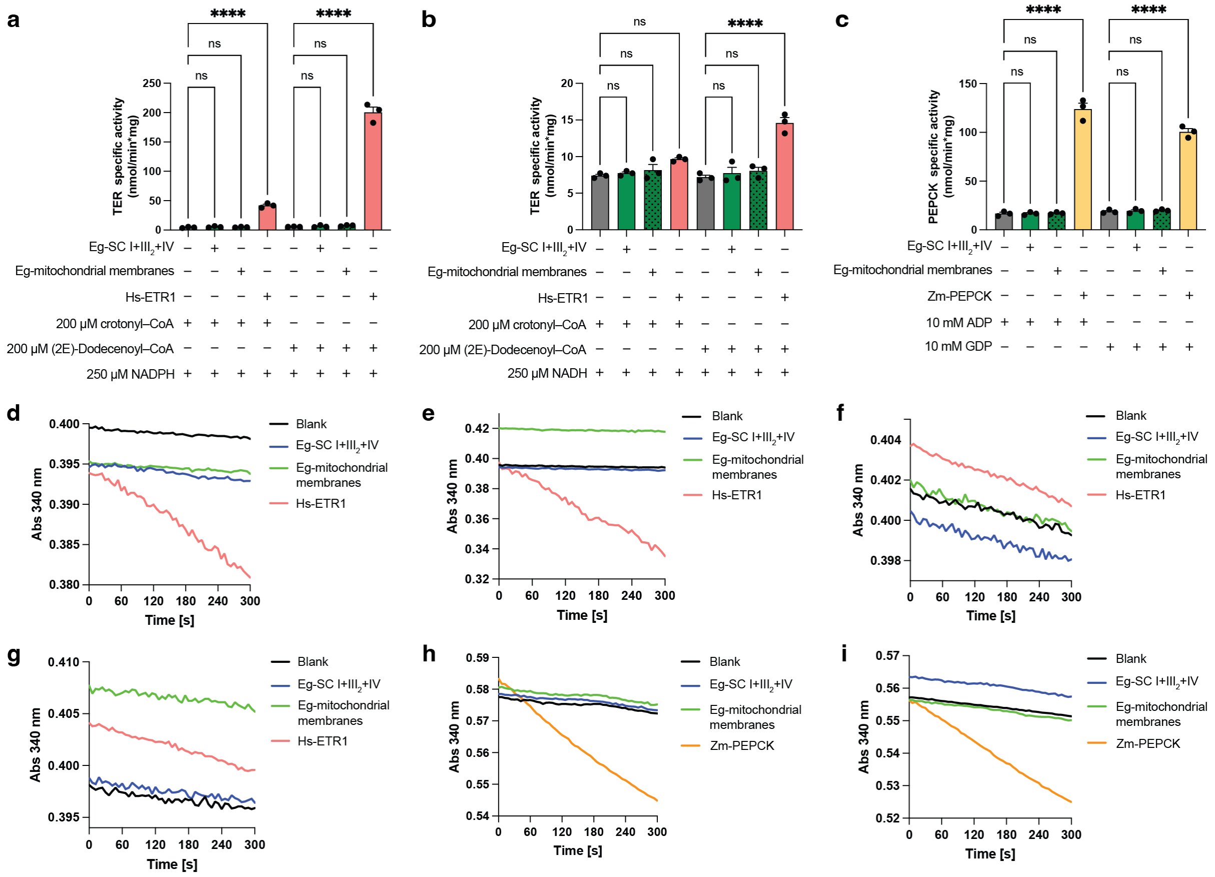

Supplementary figure 16. Spectroscopic activity assays of subunits in Eg-CI's FAS domain. a,**b**, Spectroscopic activity assays of TER by monitoring the NADPH (**a**) or NADH (**b**) oxidation at 340 nm, using Eg-SC I+III$_2$+IV (green), Eg-mitochondrial membranes (dotted green), and *H. sapiens* (Hs) ETR1 (salmon) as positive control, in the presence of crotonyl-CoA or (2E)-dodecenoyl-CoA. **c**, Spectroscopic activity assays of PEPCK by monitoring the NADH oxidation at 340 nm, using Eg-SC I+III$_2$+IV (green), Eg-mitochondrial membranes (dotted green), and *Z. mays* (Zm) PEPCK (yellow) as positive control in the presence of ADP or GDP as indicated. For (**a-c**), data are presented as mean values ± standard error of mean (SEM), n=3 biologically independent activity experiments. Statistical analysis is performed with one-way ANOVA with Tukey's multiple comparisons test. ****, $P < 0.0001$. ns, not statistically significant ($P > 0.05$). **d-i**, Kinetic curves of the TER (**d-g**) and PEPCK (**h** and **i**) activity assays. Data are presented as mean values ± standard error of mean (SEM), n=3 biologically independent activity experiments, error bars representing SEM are not shown for clarity. Source data are provided as a Source Data file.

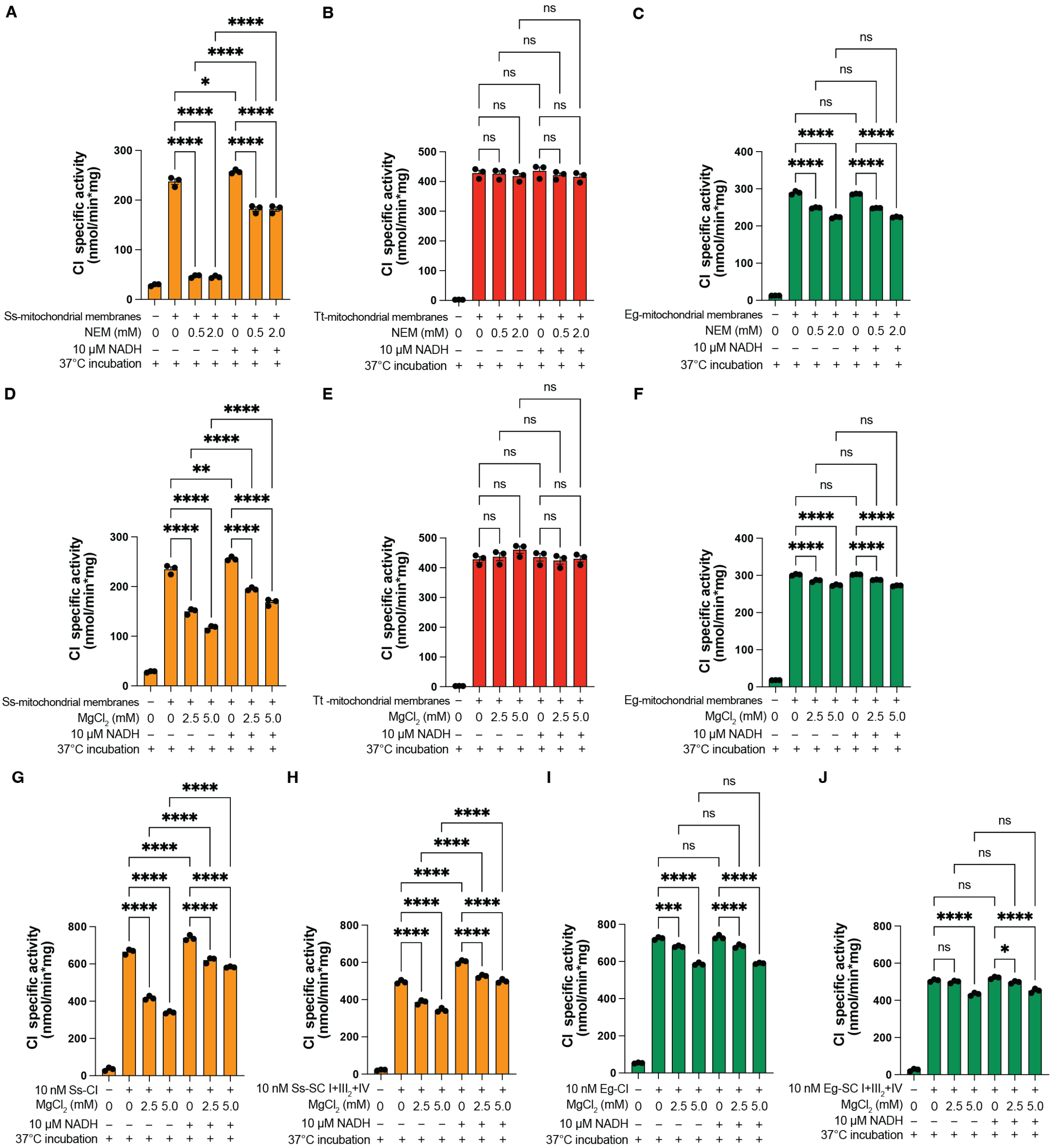

Supplementary figure 17. Additional A/D transition assays. a-c, Thermal A/D transition assays in the presence of NEM at indicated concentrations, re-activated by 10 µM NADH or equivalent buffer, using isolated mitochondrial membranes of *S. scrofa* (yellow) (**a**), *T. thermophila* (red) (**b**) and *E. gracilis* (green) (**c**) , measured spectroscopically by NADH dehydrogenase activity at 340 nm. **d-f**, A/D transition assays performed and colored as above, but in the presence of $MgCl_2$ at indicated concentrations. **g-j**, A/D transition assays in the presence of $MgCl_2$ at indicated concentrations as above, using purified proteins of *S. scrofa* (**g** and **h**) or *E. gracilis* (**i** and **j**). For (**a-j**), data are presented as mean values ± standard error of mean (SEM), n=3 biologically independent activity experiments. Statistical analysis is performed with one-way ANOVA with Tukey's multiple comparisons test. *, $P < 0.05$; **, $P < 0.01$; ***, $P < 0.001$; ****, $P < 0.0001$. ns, not statistically significant ($P > 0.05$). Source data are provided as a Source Data file.

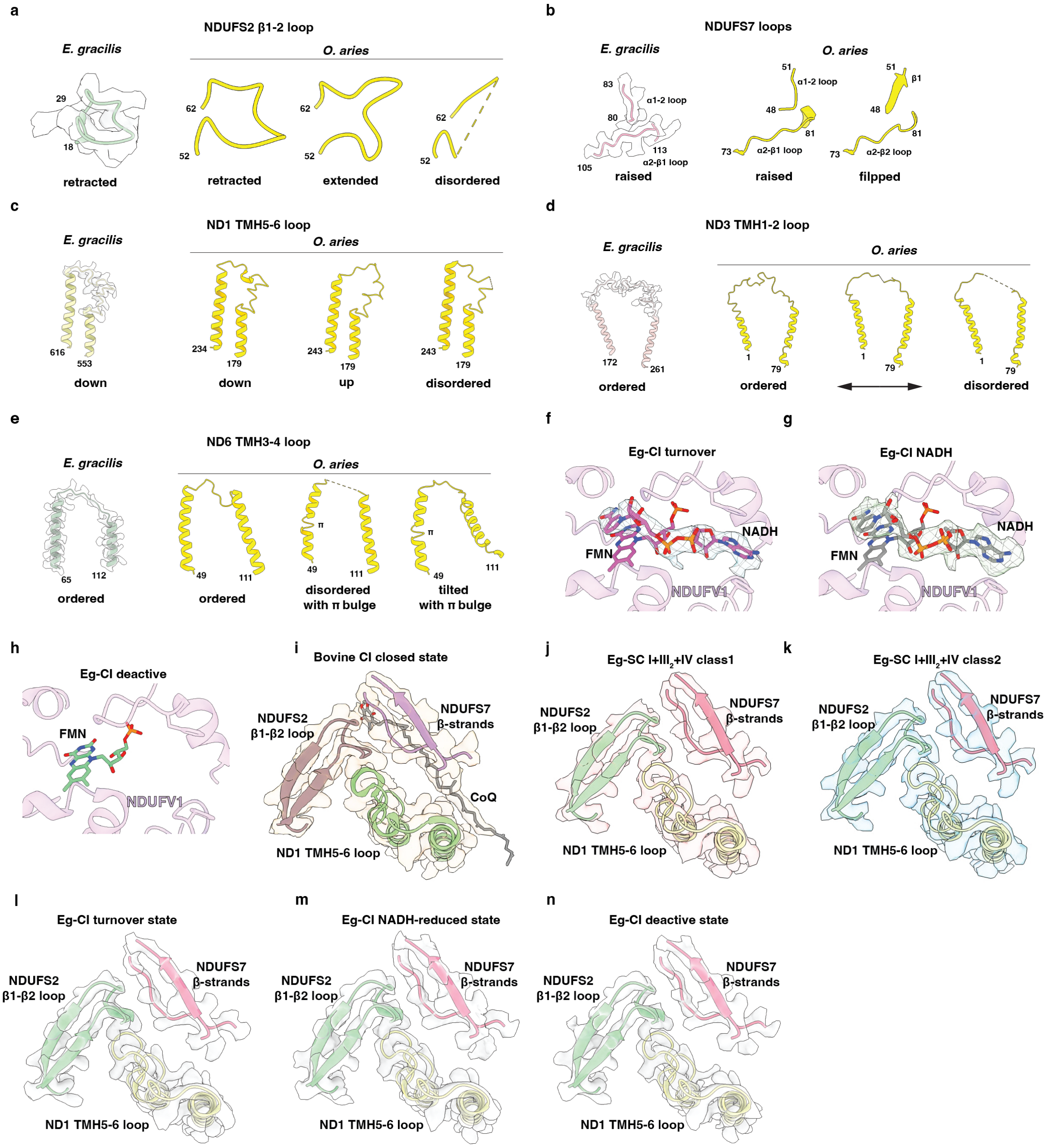

Supplementary figure 18. Local conformations of the Q tunnel loops in Eg SC I+III$_2$+IV and NADH and CoQ densities in Eg-CI maps in different states. a-e, Comparisons of the Q tunnel loop conformations between Eg- and *O. aries* SC I+III$_2$+IV, including the NDUFS2 β1-2 loop (**a**), the NDUFS7 loops (**b**), the ND1 TMH5-6 loop (**c**), the ND3 TMH1-2 loop (**d**) and the ND6 TMH3-4 loop (**e**). Structures of *O. aries* CI in the native closed (PDB 6ZKO), native open (PDB 6ZKP), rotenone closed (PDB 6ZKK), NADH open (PDB 6ZKH) and deactive open (PDB 6ZKS) states are used. The *E. gracilis* subunits are shown in cartoon and colored as in **Fig. 1e**, with local densities shown as transparent surfaces. *O. aries* subunits are shown in yellow cartoons. **f-h**, The NADH densities in Eg-CI under different catalytic conditions. NADH densities are shown as transparent surfaces for Eg-CI under turnover (**f**) and NADH reduced (**g**) states but is absent in the deactivated state (**h**). **i-n**, CoQ and Q tunnel loop densities are shown as transparent surfaces for bovine CI in lipid nanodisc (PDB 7QSK) (**i**), but is absent in Eg SC I+III$_2$+IV state 1 (**j**), state 2 (**k**) or Eg-CI under different catalytic conditions (**l-n**). Adjacent Q tunnel loops from NDUFS2, NDUFS7 and ND1 are also shown in cartoon with transparent surfaces representing densities. It is obviously that no major conformational flexibility can be observed in these Q tunnel loops from Eg-SC I+III$_2$+IV or Eg-CI states.

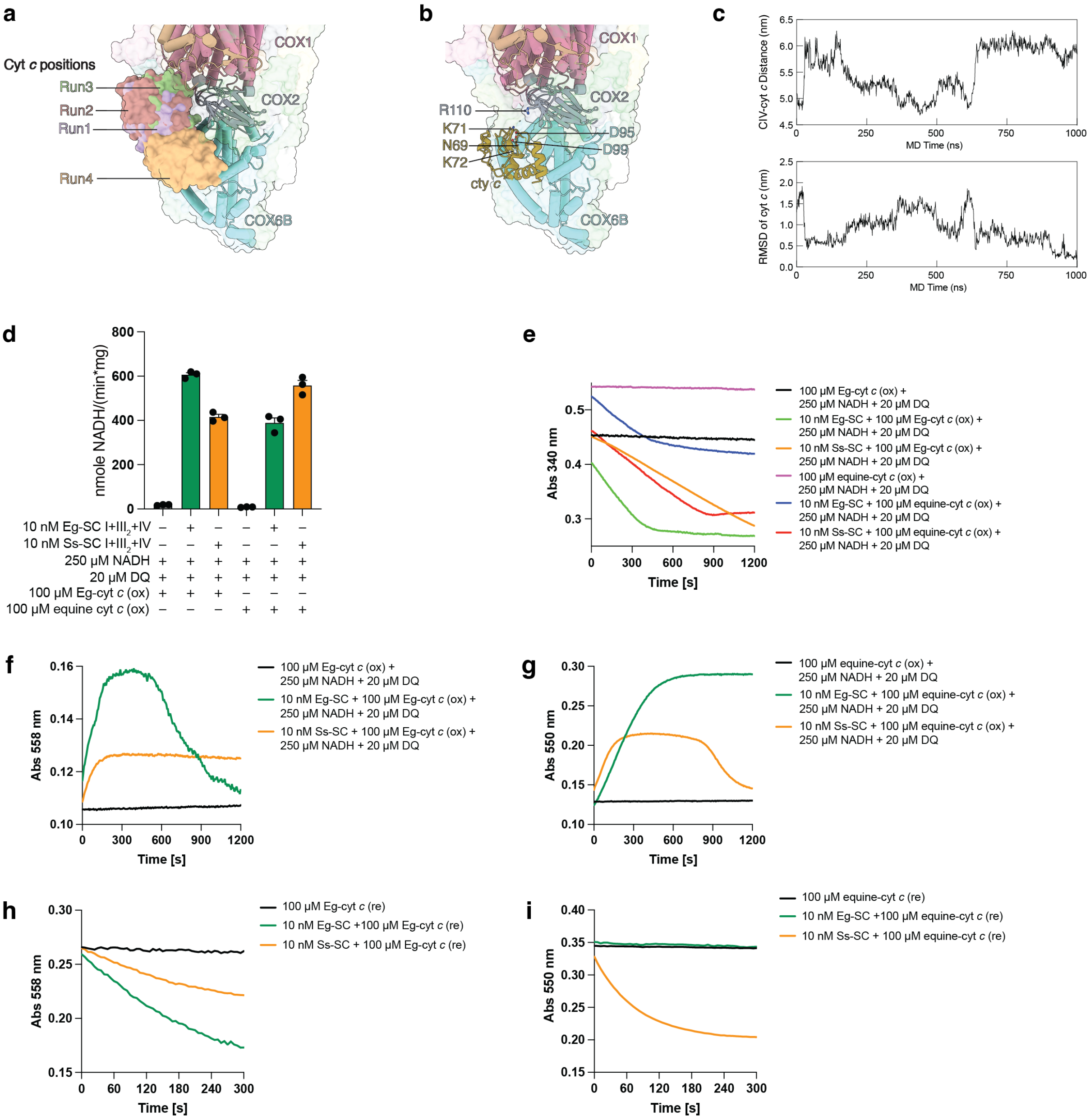

Supplementary figure 19. MD simulation and functional characterization of *E. gracilis* CIV-cyt *c* interaction. a, MD simulations (Run 1-4) of Eg-CIV+Eg-cyt *c* with different cyt *c* positions shown as colored surfaces. **b**, MD simulation Run 4 of Eg-CIV+Eg-cyt *c* with potential key interacting residues shown as sticks and colored by elements. **c**, MD trajectories (Run 4) of the center-of-mass distance between CIV and cyt *c* (upper panel) and the root-mean-square deviation (RMSD) of cyt *c* relative to the last frame of the simulation (lower panel) after aligning the entire CIV-cyt *c* complex with CIV during the 1000 ns MD simulation run. **d**, Spectroscopic assay of NADH oxidation activities of *E. gracilis* (green) and *S. scrofa* (orange) SC-I+III$_2$+IV in presence of Eg- or equine cyt *c*, monitored at 340 nm. Data are presented as mean values ± standard error of mean (SEM), n=3 biologically independent activity experiments. **e**, Kinetic curves for activity measurements in (**d**). **f**,**g**, Kinetic curves for activity measurements in **Fig. 6a** monitored at 558 nm (**f**) and 550 nm (**g**) for Eg- and equine cyt *c* respectively. Note that bars in **Fig. 6a** are calculated as the slopes of the initial linearly increasing phases in the kinetic curves. **h**,**i**, Kinetic curves for activity measurements in **Fig. 6b** monitored at 558 nm (**h**) and 550 nm (**i**) for Eg- and equine cyt *c* respectively. Note that bars in **Fig. 6b** are calculated as the slopes of the initial linearly declining phases in the kinetic curves. For (**e-i**), data are presented as mean values ± standard error of mean (SEM), n=3 biologically independent activity experiments, error bars representing SEM are not shown for clarity. Source data are provided as a Source Data file.

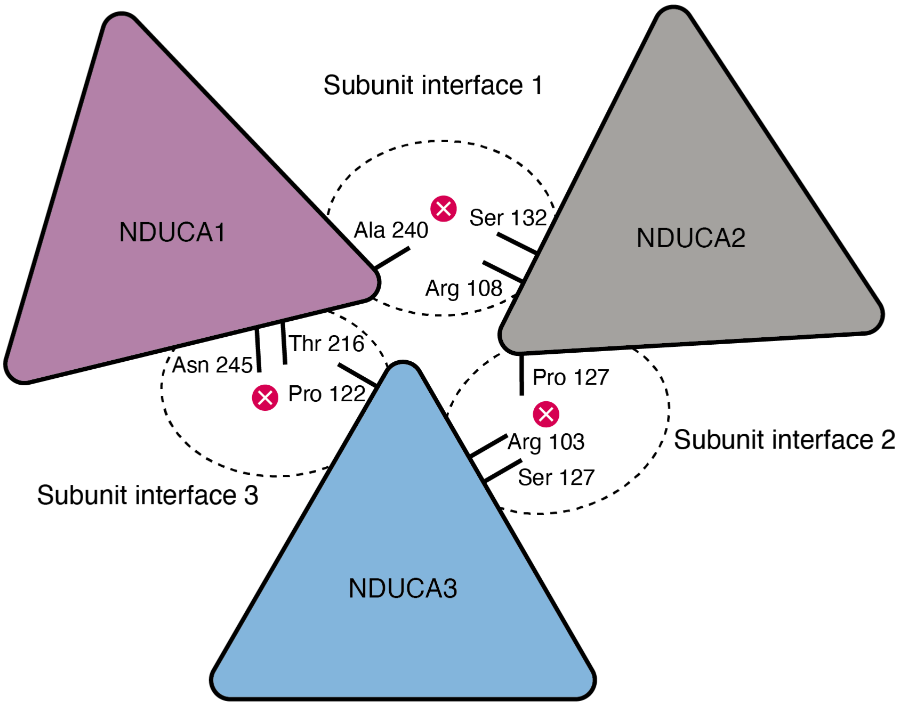

Supplementary figure 20. Schematic representation of subunit interfaces in Eg-CI's γCA domain. The three γCA subunits NDUCA1 (purple), NDUCA2 (gray) and NDUCA3 (blue) are represented as cartoon triangles. Homologous residues for the Zn^{2+}-coordinating residues of CamH γCA as per sequence alignment, are shown. Red crosses indicate absence of coordinated Zn^{2+} density.

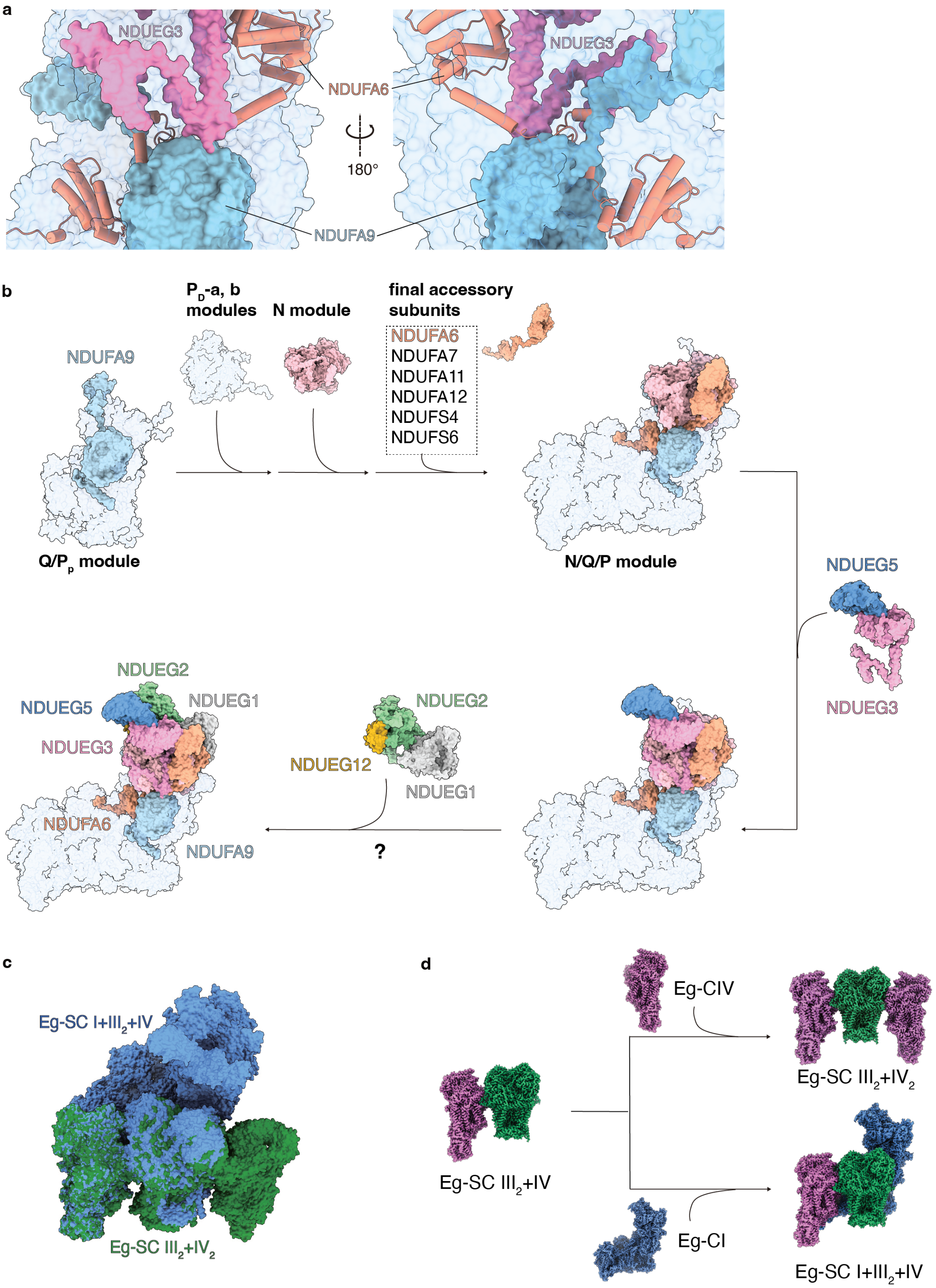

Supplementary figure 21. The proposed assembly procedures of *E. gracilis* CI and supercomplexes. **a**, Structural relations between NDUEG3, NDUFA6 and NDUFA9. Note that the NDUFA6 helix is overlaid by the CT helix of NDUEG3 and the central β sheet of NDUFA9. **b**, Proposed scheme of the *E. gracilis* CI assembly. Note that the NDUEG3-5 heterodimer is assembled to Eg-CI after N module

attachment, which could be followed by the assembly of the rest of the FAS domain subunits. The subunits and modules are shown as solid surfaces. **c,** Alignment of Eg-SC III_2+IV_2 (green surface) to Eg-SC I+III_2+IV (blue surface) by the SC III_2+IV. **d**, Two possible pathways for supercomplex assembly starting from Eg-SC III_2+IV.

Supplementary Table 1. Cryo-EM and modelling statistics of Eg-supercomplexes

	Dataset 1<br>Eg-SC CI+III_2+IV<br>(EMDB-35720)<br>(PDB 8IUF)	Dataset 1<br>Eg-SC $CIII_2$+IV_2<br>(EMDB-35723)<br>(PDB 8IUJ)
**Data collection and processing**	**subset 1**	**subset 2**
Magnification	105,000	105,000
Voltage (kV)	300	300
Electron exposure (e–/Å^2)	51.51	51.51
Defocus range (μm)	-0.6 to -1.8	-0.6 to -1.8
Pixel size (Å)	1.20	1.20
Symmetry imposed		
Initial particle images (no.)	9807	9807
Final particle images (no.)		
Map resolution (Å)<br>FSC threshold	CI PA: 2.73<br>CI MAp: 2.72<br>CI MAd: 2.69<br>$CIII_2$: 2.77<br>CIV: 2.76	$CIII_2$: 2.85<br>CIV-L: 2.89<br>CIV-R: 3.14
Map resolution range (Å)	2.69-2.77	2.85-3.14
**Refinement**		
Initial model used (PDB code)		
Model resolution (Å)		
FSC threshold	0.5	0.5
Model resolution range (Å)		
Map sharpening *B* factor (Å^2)	CI PA: 107.3<br>CI MAp:107.1<br>CI MAd: 106.7<br>$CIII_2$: 112.9<br>CIV: 115.1	$CIII_2$: 97<br>CIV-L: 97.7<br>CIV-R: 102.1
Model composition		
Non-hydrogen atoms	185137	100747
Protein residues	22245	12090
Ligands	115	73
B factors (Å^2)		
Protein	51.36	432.26
Ligand	22.76	50.14
R.m.s. deviations		
Bond lengths (Å)	0.006	0.005
Bond angles (°)	0.573	0.555
Validation		
MolProbity score	1.35	1.40
Clashscore	3.02	3.42
Poor rotamers (%)	0.78	0.85
Ramachandran plot		
Favored (%)	96.25	96.09
Allowed (%)	3.75	3.90
Disallowed (%)	0.01	0.02

Supplementary Table 2. Cryo-EM and modelling statistics of Eg-CI under different states

	Dataset 2<br>Eg-CI, deactive<br>(EMDB-36107)<br>(PDB 8J9H)	Dataset 3<br>Eg-CI, turnover<br>(EMDB-36108)<br>(PDB 8J9I)	Dataset 4<br>Eg-CI, NADH<br>(EMDB-36109)<br>(PDB 8J9J)
**Data collection and processing**	**Dataset 2**	**Dataset 3**	**Dataset 4**
Magnification	130,000	130,000	130,000
Voltage (kV)	300	300	300
Electron exposure (e–/Å^2)	61.5	61.5	61.5
Defocus range (μm)	-0.8 to -2.0	-0.8 to -2.0	-0.8 to -2.0
Pixel size (Å)	0.93	0.93	0.93
Symmetry imposed			
Initial particle images (no.)	2702	6758	2949
Final particle images (no.)			
Map resolution (Å)	CI PA: 3.07	CI PA: 2.82	CI PA: 2.97
FSC threshold	CI MAp: 3	CI MAp: 2.87	CI MAp: 2.93
	CI MAd: 3.03	CI MAd: 2.89	CI MAd: 2.94
Map resolution range (Å)	3-3.07	2.82-2.89	2.93-2.97
**Refinement**			
Initial model used (PDB code)			
Model resolution (Å)			
FSC threshold	0.5	0.5	0.5
Model resolution range (Å)			
Map sharpening *B* factor (Å^2)	CI PA: 77.9	CI PA: 87.1	CI PA: 77
	CI MAp: 80.1	CI MAp: 87.6	CI MAp: 78.5
	CI MAd: 82.4	CI MAd: 91.5	CI MAd: 82
Model composition			
Non-hydrogen atoms	113596	113971	113640
Protein residues	13647	13686	13647
Ligands	64	65	65
B factors (Å^2)			
Protein	51.64	50.39	50.40
Ligand	23.32	22.11	22.11
R.m.s. deviations			
Bond lengths (Å)	0.004	0.004	0.006
Bond angles (°)	0.552	0.518	0.614
Validation			
MolProbity score	1.8	1.57	1.92
Clashscore	7.15	5.53	8.10
Poor rotamers (%)	1.47	1.18	1.5
Ramachandran plot			
Favored (%)	95.94	96.61	95.09
Allowed (%)	4.04	3.38	4.87
Disallowed (%)	0.02	0.01	0.04

Supplementary Table 3. Eg-SC I+III$_2$+IV model summary

Subunit Name	Subunit identifier	Annotation	Chain ID	# Total residues	Atomic Residues	% Atomic	TMH	Ligands	Lipids	Notes
**CI Peripheral arm core subunits**										
NDUFV1	comp62912_c0 _seq. 6	NADH dehydrogenase [ubiquinone] flavoprotein 1, mitochondrial	V1	526	18-521	95.8%	0	1 × 4Fe4S, 1 × FMN		
NDUFV2	EG_transcript_19472 sga_contig_466476 sga_contig_302717	NADH dehydrogenase [ubiquinone] flavoprotein 2	V2	NA	5-225	NA	0	1 × 2Fe2S		Poly-UNK: 1-4
NDUFS1A	comp61469_c0_seq1 _c	NADH dehydrogenase/NADH: ubiquinone oxidoreductase 75 kDa subunit	1A	385	25-376	91.4%	0	1 × 2Fe2S, 2 × 4Fe4S		
NDUFS1B	EG_transcript_7035	16S rRNA (cytosine(1402)-N(4))-methyltransferase	1B	527	3-527	99.6%	0			
NDUFS2	EG_transcript_13592	NADH dehydrogenase subunit D; NADH:ubiquinone oxidoreductase 49 kDa subunit 7	S2	395	2-395	99.7%	0			N3,N4-dimethylar ginne
NDUFS3	comp62960_c0 _seq11	NADH dehydrogenase subunit C	S3	277	26-273	89.5%	0			
NDUFS7	EG_transcript_32395	NADH dehydrogenase [ubiquinone] Fe-S protein 7	S7	NA	7-202	NA	0	1 × 4Fe4S		Poly-UNK: 203-207
NDUFS8	comp54309_c0_seq4 _c	NADH dehydrogenase subunit I	S8	212	30-211	85.8%	0	2 × 4Fe4S		
CI Membrane arm core subunits										
ND1	sga_contig_684056	NADH-ubiquinone oxidoreductase chain 1	N1	670	361-670	46.3%	8		2 × PC1	

ND2A	sga_contig_881214	Ymf65	N2	300	4-299	98.7%	11		1 × PC1	
ND2B	sga_contig_1967065	NADH dehydrogenase subunit 2	2B	NA	3-115	NA	3			Poly-UNK: 116-142
ND3	sga_contig_1876491	NADH-ubiquinone oxidoreductase chain 3	N3	NA	172-292	NA	3		1 × PC1	
ND4	sga_contig_171091	NADH-ubiquinone oxidoreductase chain 4L	N4	478	1-478	100%	14	1×U10	1 × CDL, 2 × PC1, 1 ×3PE	
ND4L	EG_transcript_32304	NADH dehydrogenase subunit 5	4L	171	61-168	63.2%	3			
ND5	sga_contig_1876491	NADH dehydrogenase subunit 5	N5	584	1-584	100%	15		3 × PC1, 2 × CDL, 1 × 3PE	
ND6	sga_contig_1876491	ADH-ubiquinone oxidoreductase chain 6	N6	NA	18-171	NA	5			
**CI Peripheral arm accessory subunits**										
NDUFA2	EG_transcript_28989	NADH dehydrogenase [ubiquinone] 1 alpha subcomplex subunit 2	A2	193	2-193	99.5%	0			
NDUFA5	EG_transcript_24541	NADH dehydrogenase [ubiquinone] 1 alpha subcomplex subunit 5	A5	184	21-174	83.7%	0			
NDUFA6	EG_transcript_12102	NADH dehydrogenase [ubiquinone] 1 alpha subcomplex subunit 6	A6	434	12-434	97.5%	0			
NDUFA7	EG_transcript_32205	NADH dehydrogenase [ubiquinone] 1 alpha subcomplex subunit 7	A7	136	1-136	100%	0			

NDUFA9	comp54702_c0_seq3	NADH dehydrogenase [ubiquinone] 1 alpha subcomplex subunit 9	A9	489	6-489	99.0%	0	1 × NDP	2 × PC1	
NDUFA12	EG_transcript_19840	NADH dehydrogenase [ubiquinone] 1 alpha subcomplex subunit 12	AL	281	17-281	94.3%	0		1 × PC1, 3 × CDL	
NDUFS4	EG_transcript_30365	NADH dehydrogenase [ubiquinone] iron-sulfur protein 4	S4	NA	16-205	NA	0			
NDUFS6	comp55416_c0_seq4	NADH dehydrogenase [ubiquinone] iron-sulfur protein 6	S6	147	1-147	100%	0	1 × Zn^{2+}		
NDUEG1	comp63840_c0_seq1	Adenylate forming domain, Class I	E1	NA	26-475	NA	0			
NDUEG2	sga_contig_620624 EG_transcript_10882	NADH dehydrogenase [ubiquinone] Euglenozoa-specific subunit 12	E2	NA	2-467	NA	0			
NDUEG3	comp63125_c0_seq1	2-enoyl thioester reductase (N,M)	E3	433	2-433	99.8%	0			
NDUEG4	comp59654_c0_seq4	NADH dehydrogenase [ubiquinone] subunit	E4	368	18-368	95.4%	0		1 × PC1	
NDUEG5	EG_transcript_18307	oxidoreductase, putative	E5	289	1-29/32-75/79-234/238-289	96.9%	0			
NDUEG6	comp60945_c0_seq5_c	DnaJ molecular chaperone homology domain	E6	371	30-371	92.2%	0		2 × CDL	
NDUEG12	EG_transcript_41155	NADH dehydrogenase [ubiquinone] 1 alpha/beta subcomplex 1	EC	101	17-101	84.2%	0			
**CI Membrane arm accessory subunits**										

NDUFA1	comp54200_c0_seq1	NADH dehydrogenase [ubiquinone] 1 alpha subcomplex subunit 1	A1	141	2-138	97.2%	1		2 × PC1	
NDUFA3	comp51611_c0_seq1_cut	NADH dehydrogenase [ubiquinone] 1 alpha subcomplex subunit 3	A3	125	1-124	99.2%	1		1 × CDL	
NDUFA8	EG_transcript_27781	NADH dehydrogenase [ubiquinone] 1 alpha subcomplex subunit 8	A8	223	1-223	100%	0			
NDUFA11	comp58177_c0_seq3	NADH dehydrogenase [ubiquinone] 1 alpha subcomplex subunit 11	AN	287	1-287	100%	5		1 × 3PE, 1 × PC1	
NDUFA13	comp59406_c0_seq3	NADH dehydrogenase [ubiquinone] 1 alpha subcomplex subunit 13	AM	198	15-198	92.9%	1		3 × CDL, 2 × PC1	
NDUFB2	comp54442_c0_seq1	NADH dehydrogenase [ubiquinone] 1 beta subcomplex subunit 2	B2	NA	41-145	NA	1			
NDUFB3	sga_contig_549447	NADH dehydrogenase [ubiquinone] 1 beta subcomplex subunit 3	B3	NA	2-34	NA	1		1 × CDL	Poly-UNK: 35-62
NDUFB4	EG_transcript_36579	NADH dehydrogenase [ubiquinone] 1 beta subcomplex subunit 4	B4	171	1-171	100%	1			
NDUFB5	EG_transcript_44892	NADH dehydrogenase [ubiquinone] 1 beta subcomplex subunit 5	B5	NA	1-36/43-140	NA	1		2 × PC1, 1 × CDL	Poly-UNK: 37-42

NDUFB6	sga_contig_1395648<br>sga_contig_881622	NADH dehydrogenase [ubiquinone] 1 beta subcomplex subunit 6	B6	NA	1-91	NA	1			
NDUFB7	sga_contig_1444062	NADH dehydrogenase [ubiquinone] 1 beta subcomplex subunit 7	B7	NA	1-97	NA	0			
NDUFB8	EG_transcript_27273	NADH dehydrogenase [ubiquinone] 1 beta subcomplex subunit 8	B8	176	25-171	83.5%	1			
NDUFB9	comp53986_c0_seq1_cut	NADH dehydrogenase [ubiquinone] 1 beta subcomplex subunit 9	B9	158	8-158	95.6%	0			
NDUFB10	comp54566_c0_seq2	NADH dehydrogenase [ubiquinone] 1 beta subcomplex subunit 10	BL	144	1-144	100%	0			
NDUFB11	comp51117_c0_seq1<br>sga_contig_881622	NADH dehydrogenase [ubiquinone] 1 beta subcomplex subunit 11	BM	NA	1-100	NA	1			Poly-UNK: 101-112
NDUFC2	comp52747_c0_seq1_cut	NADH dehydrogenase [ubiquinone] 1 subunit C2	C4	185	1-183	98.9%	1		2 × CDL,<br>1 × PC1	
NDUFAB1-α	gnl|Egra|Contig2592	Acyl carrier protein, mitochondrial	AB	134	47-134	65.7%	0	1 × ZMP		
NDUFAB1-β	gnl|Egra|Contig2076	Acyl carrier protein, mitochondrial	AC	134	43-134	68.7%	0	1 × ZMP		
NDUFS5	EG_transcript_58467	NADH dehydrogenase [ubiquinone] iron-sulfur protein 5	S5	122	1-111	91.0%	0			Poly-UNK: 112-122
NDUFX	comp52123_c0_seq2_gi|125990644	Glyceraldehyde 3-phosphate dehydrogenase, C-terminal domain	FX	325	88-324	72.9%	0			

NDUCA1	comp62122_c0_seq3_c	NADH dehydrogenase [ubiquinone] subunit; CA1 (gamma carbonic anhydrase)	G1	436	11-436	97.7%	0		1 × 3PE	
NDUCA2	comp48089_c0_seq3_cut	NADH dehydrogenase [ubiquinone] subunit; CA2 (gamma carbonic anhydrase)	G2	267	2-237	88.4%	0			
NDUCA3	EG_transcript_22381 EG_transcript_46696 sga_contig_833122_ 5'-3' Frame 3	NADH dehydrogenase [ubiquinone] subunit; CA3 (gamma carbonic anhydrase)	G3	NA	8-261	NA	0			Poly-UNK: 1-7
NDUEG7	sga_contig_833122_ 5'-3' Frame 3 EG_transcript_22716	LRAT-like domain-containing protein	E7	NA	1-246	NA	3	1 × Zn^{2+}	1 × CDL	
NDUEG8	comp47716_c0_seq2_cut	hypothetical protein BRAFLDRAFT_83552	E8	205	1-205	100%	1		4 × PC1	
NDUEG9	EG_transcript_26334	sjchgc05781 protein	E9	178	1-165	92.7%	4		1 × PC1	
NDUEG10	EG_transcript_65450	Alpha-2-antiplasmin	EA	126	3-99	77.0%	1		2 × CDL	Poly-UNK: 100-126
NDUEG11	sga_contig_1835241 sga_contig_1398522	Unknown protein	EB	NA	28-101	NA	0			Poly-UNK: 1-27
NDUEG13	comp41364_c0_seq3	Unknown protein	ED	151	14-151	91.4%	1		1 × PC1	
U1	-	-	U1	NA	1-12	NA	0			Poly-UNK: 1-12
U2	-	-	U2	NA	1-12	NA	0			Poly-UNK: 1-12
**CIII**										
MPP-α	comp60854_c0_seq1_cut	Cytochrome b-c1 complex subunit 2	QB	474	20-474	96.0%	0			

MPP-β	comp63646_c0_seq8 _cut	Cytochrome b-c1 complex subunit 1	QA	479	1-476	99.4%	0			
COB	sga_contig_914092	Cytochrome b	QC	368	2-365	98.9%	8	2×b-heme	2 × CDL, 1 × PC1	
CYC1	comp49373_c0_seq3 _cut	Cytochrome c1, heme protein	QD	244	3-243	98.8%	1	1× c-heme	1 × CDL, 1 × PC1	
UQCRFS1	comp57996_c0_seq3 _cut	Cytochrome b-c1 complex subunit Rieske	QE	252	17-247	91.7%	1	1× 2Fe2S	1 × CDL, 1 × PC1, 1 × PX2	
UQCRH	gnl|Egra|109779475	Cytochrome b-c1 complex subunit 6	QF	NA	9-72	NA	0			
UQCRB	comp51517_c0_seq3 _cut	Cytochrome b-c1 complex subunit 7	QG	228	1-228	100%	0			
UQCRQ	sga_contig_617119 sga_contig_1173247	Cytochrome b-c1 complex subunit 8	QH	NA	2-86	NA	1			
UQCR9	sga_contig_1991138	Cytochrome b-c1 complex subunit 9	QI	NA	1-29	NA	1			Poly-UNK: 30-70
UQCR10	comp57617_c0_seq1 _cut	Cytochrome b-c1 complex subunit 10	QJ	154	6-154	96.8%	1		1 × CDL, 1 × PX2	
UQCREG1	gnl|Egra|Contig585	ubiquinol-cytochrome c reductase subunit	QK	100	31-91	61%	1			
MPP-α	comp60854_c0_seq1 _cut	Cytochrome b-c1 complex subunit 2	Qb	474	52-474	89.2%	0			
MPP-β	comp63646_c0_seq8 _cut	Cytochrome b-c1 complex subunit 1	Qa	479	1-476	99.4%	0			
COB	sga_contig_914092	Cytochrome b	Qc	368	2-365	98.9%	8	2×b-heme	1 × CDL	
CYC1	comp49373_c0_seq3 _cut	Cytochrome c1, heme protein	Qd	244	3-243	98.8%	1	1× c-heme	1 × CDL, 1 × PC1	

UQCRFS1	comp57996_c0_seq3_cut	Cytochrome b-c1 complex subunit Rieske	Qe	252	17-247	91.7%	1	1× 2Fe2S	2 × CDL, 1 × PC1	
UQCRH	gnl|Egra|109779475	Cytochrome b-c1 complex subunit 6	Qf	NA	9-72	NA	0			
UQCRB	comp51517_c0_seq3_cut	Cytochrome b-c1 complex subunit 7	Qg	228	1-228	100%	0		1 × PC1	
UQCRQ	sga_contig_617119 sga_contig_1173247	Cytochrome b-c1 complex subunit 8	Qh	NA	2-86	NA	1			
UQCR9	sga_contig_1991138	Cytochrome b-c1 complex subunit 9	Qi	NA	1-29	NA	1			Poly-UNK: 30-70
UQCR10	comp57617_c0_seq1_cut	Cytochrome b-c1 complex subunit 10	Qj	154	6-154	96.8%	1		1 × CDL, 2 × PC1	
UQCREG1	gnl|Egra|Contig585	ubiquinol-cytochrome c reductase subunit	Qk	100	31-91	61%	1			
**CIV**										
**CIV Core subunits**										
COX1	sga_contig_1208836	Cytochrome c oxidase subunit 1	C1	495	1-495	100%	12	2×a-heme, 1 × Cu_B, 1 ×Mg^{2+}	3 × 3PE, 1 × PC1, 1 × CDL	
COX2	gnl|Egra|5053115	Cytochrome c oxidase subunit 2	C2	196	1-196	100%	2	2 × Cu_A		
COX3	sga_contig_1132792	Cytochrome c oxidase subunit 3	C3	161	1-161	100%	4		1 × PC1	
**CIV Conserved subunits**										
COX4	comp53543_c0_seq2	Cytochrome c oxidase subunit 4	DC	179	13-179	93.3%	1			
COX5B-2	comp54722_c0_seq3_cut	Cytochrome c oxidase subunit 5b-2	5B	174	18-174	90.2%	0	1 × Zn^{2+}		
COX6A	EG_transcript_36110	Cytochrome c oxidase subunit 6a	6A	112	1-91	81.3%	1			
COX6B-1	comp54364_c0_seq4	Cytochrome c oxidase subunit 6b-1	6B	287	1-282	98.3%	0			

COX5C	gnl|Egra|Contig1435	Cytochrome c oxidase subunit 5c	5C	NA	12-207	NA	1				
COX7A	comp47102_c0_seq2<br>_cut	Cytochrome c oxidase subunit 7a	7A	178	1-47/61-<br>178	92.1%	1		1 × PC1,<br>1 × CDL	
COX7C	comp55710_c0_seq1<br>_cut	Cytochrome c oxidase subunit 7c	7C	171	21-171	88.3%	1			
**CIV Specific subunits**										
COXEG1	comp54737_c0_seq2	MFS transporter	4A	246	43-246	93.6%	3		2 × PC1	
COXEG2	comp50120_c1_seq1<br>_cut	hypothetical protein [*Monosiga brevicollis* MX1]	4C	139	16-138	88.5%	1		1 × PX2	
COXEG3	gnl|Egra|Contig2618	hypothetical protein<br>HELRODRAFT_75559	4D	174	2-174	99.4%	1		2 × 3PE	
COXEG4	EG_transcript_26565	Unknown protein	4E	165	2-161	97.0%	1		1 × PC1	
COXEG5	gnl|Egra|Contig715<br>sga_contig_1482041	Unknown protein	4F	NA	1-75	NA	1		1 × S12	
COXEG6	comp55436_c0_seq3<br>_cut	cytochrome c oxidase assembly<br>Euglenozoa-specific subunit 4	4G	315	19-315	94.3%	0			
COXEG7	gi|109781798_cut	cytochrome oxidase subunit 5	4H	221	17-221	92.8%	0			
COXEG8	comp57506_c0_seq2<br>_cut	Unknown protein	4I	274	10-274	96.7%	0			
COXEG9	comp53374_c0_seq4	Unknown protein	4J	88	1-88	100%	0			

Supplementary Table 4. Eg-SC III_2+IV_2 model summary

Subunit Name	Subunit identifier	Annotation	Chain ID	# Total residues	Atomic Residues	% Atomic	TMH	Ligands	Lipids	Notes
**CIII**										
MPP-α	comp60854_c0_seq1_cut	Cytochrome b-c1 complex subunit 2	QB	474	20-474	96.0%	0			
MPP-β	comp63646_c0_seq8_cut	Cytochrome b-c1 complex subunit 1	QA	479	1-476	99.4%	0			
COB	sga_contig_914092	Cytochrome b	QC	368	2-365	98.9%	8	2×b-heme	2 × CDL, 1 × PC1	
CYC1	comp49373_c0_seq3_cut	Cytochrome c1, heme protein	QD	244	3-243	98.8%	1	1× c-heme	1 × CDL, 1 × PC1	
UQCRFS1	comp57996_c0_seq3_cut	Cytochrome b-c1 complex subunit Rieske	QE	252	17-247	91.7%	1	1× 2Fe2S	1 × CDL, 1 × PC1, 1 × PX2	
UQCRH	gnl|Egra|109779475	Cytochrome b-c1 complex subunit 6	QF	NA	9-72	NA	0			
UQCRB	comp51517_c0_seq3_cut	Cytochrome b-c1 complex subunit 7	QG	228	1-228	100%	0			
UQCRQ	sga_contig_617119 sga_contig_1173247	Cytochrome b-c1 complex subunit 8	QH	NA	2-86	NA	1			
UQCR9	sga_contig_1991138	Cytochrome b-c1 complex subunit 9	QI	NA	1-29	NA	1			Poly-UNK: 30-70
UQCR10	comp57617_c0_seq1_cut	Cytochrome b-c1 complex subunit 10	QJ	154	6-154	96.8%	1		1 × CDL, 1 × PX2	
UQCREG1	gnl|Egra|Contig585	ubiquinol-cytochrome c reductase subunit	QK	100	31-91	61%	1			

MPP-α	comp60854_c0_seq1_cut	Cytochrome b-c1 complex subunit 2	Qb	474	20-474	96.0%	0			
MPP-β	comp63646_c0_seq8_cut	Cytochrome b-c1 complex subunit 1	Qa	479	1-476	99.4%	0			
COB	sga_contig_914092	Cytochrome b	Qc	368	2-365	98.9%	8	2×b-heme	1 × CDL, 1 × PC1	
CYC1	comp49373_c0_seq3_cut	Cytochrome c1, heme protein	Qd	244	3-243	98.8%	1	1× c-heme	1 × CDL, 1 × PC1	
UQCRFS1	comp57996_c0_seq3_cut	Cytochrome b-c1 complex subunit Rieske	Qe	252	17-247	91.7%	1	1× 2Fe2S	2 × CDL, 1 × PC1	
UQCRH	gnl|Egra|109779475	Cytochrome b-c1 complex subunit 6	Qf	NA	9-72	NA	0			
UQCRB	comp51517_c0_seq3_cut	Cytochrome b-c1 complex subunit 7	Qg	228	1-228	100%	0		1 × PC1	
UQCRQ	sga_contig_617119 sga_contig_1173247	Cytochrome b-c1 complex subunit 8	Qh	NA	2-86	NA	1			
UQCR9	sga_contig_1991138	Cytochrome b-c1 complex subunit 9	Qi	NA	1-29	NA	1			Poly-UNK: 30-70
UQCR10	comp57617_c0_seq1_cut	Cytochrome b-c1 complex subunit 10	Qj	154	6-154	96.8%	1		1 × CDL, 2 × PC1	
UQCREG1	gnl|Egra|Contig585	ubiquinol-cytochrome c reductase subunit	Qk	100	31-91	61%	1			
**CIV**										
**CIV Core subunits**										
COX1	sga_contig_1208836	Cytochrome c oxidase subunit 1	C1	495	1-495	100%	12	2×a-heme, 1 × Cu_B, 1 ×Mg^{2+}	3 × 3PE, 2 × PC1, 1 × CDL	
COX2	gnl|Egra|5053115	Cytochrome c oxidase subunit 2	C2	196	1-196	100%	2	2 × Cu_A		

COX3	sga_contig_1132792	Cytochrome c oxidase subunit 3	C3	161	1-161	100%	4		1 × PC1	
**CIV Conserved subunits**										
COX4	comp53543_c0_seq2	Cytochrome c oxidase subunit 4	DC	179	13-179	93.3%	1			
COX5B-2	comp54722_c0_seq3_cut	Cytochrome c oxidase subunit 5b-2	5B	174	18-174	90.2%	0	1 × Zn^{2+}		
COX6A	EG_transcript_36110	Cytochrome c oxidase subunit 6a	6A	112	1-111	99.1%	1			
COX6B-1	comp54364_c0_seq4	Cytochrome c oxidase subunit 6b-1	6B	287	1-282	98.3%	0			
COX5C	gnl|Egra|Contig1435	Cytochrome c oxidase subunit 5c	5C	NA	12-207	NA	1			
COX7A	comp47102_c0_seq2_cut	Cytochrome c oxidase subunit 7a	7A	178	1-47/61-178	92.1%	1		1 × PC1, 1 × CDL	
COX7C	comp55710_c0_seq1_cut	Cytochrome c oxidase subunit 7c	7C	171	21-171	88.3%	1			
**CIV Specific subunits**										
COXEG1	comp54737_c0_seq2	MFS transporter	4A	246	43-246	93.6%	3		2 × PC1	
COXEG2	comp50120_c1_seq1_cut	hypothetical protein [Monosiga brevicollis MX1]	4C	139	16-138	88.5%	1		1 × PX2	
COXEG3	gnl|Egra|Contig2618	hypothetical protein HELRODRAFT_75559	4D	174	2-174	99.4%	1		2 × 3PE	
COXEG4	EG_transcript_26565	Unknown protein	4E	165	2-161	97.0%	1		1 × PC1	
COXEG5	gnl|Egra|Contig715 sga_contig_1482041	Unknown protein	4F	NA	1-75	NA	1		1 × S12	
COXEG6	comp55436_c0_seq3_cut	cytochrome c oxidase assembly Euglenozoa-specific subunit 4	4G	315	19-315	94.3%	0			
COXEG7	gi|109781798_cut	cytochrome oxidase subunit 5	4H	221	17-221	92.8%	0			
COXEG8	comp57506_c0_seq2_cut	Unknown protein	4I	274	10-274	96.7%	0			

COXEG9	comp53374_c0_seq4	Unknown protein	4J	88	1-88	100%	0			
**CIV**										
**CIV Core subunits**										
COX1	sga_contig_1208836	Cytochrome c oxidase subunit 1	c1	495	1-495	100%	12	2×a-heme, 1 × Cu_B, 1 ×Mg^{2+}	3 × 3PE, 1 × PC1, 1 × CDL	
COX2	gnl|Egra|5053115	Cytochrome c oxidase subunit 2	c2	196	1-196	100%	2	2 × Cu_A		
COX3	sga_contig_1132792	Cytochrome c oxidase subunit 3	c3	161	1-161	100%	4		1 × PC1	
**CIV Conserved subunits**										
COX4	comp53543_c0_seq2	Cytochrome c oxidase subunit 4	dc	179	13-179	93.3%	1		1 × PC1	
COX5B-2	comp54722_c0_seq3_cut	Cytochrome c oxidase subunit 5b-2	5b	174	18-174	90.2%	0	1 × Zn^{2+}		
COX6A	EG_transcript_36110	Cytochrome c oxidase subunit 6a	6a	112	1-91	81.3%	1			
COX6B-1	comp54364_c0_seq4	Cytochrome c oxidase subunit 6b-1	6b	287	1-282	98.3%	0			
COX5C	gnl|Egra|Contig1435	Cytochrome c oxidase subunit 5c	5c	NA	12-207	NA	1			
COX7A	comp47102_c0_seq2_cut	Cytochrome c oxidase subunit 7a	7a	178	1-47/61-178	92.1%	1		1 × PC1, 1 × CDL	
COX7C	comp55710_c0_seq1_cut	Cytochrome c oxidase subunit 7c	7c	171	21-171	88.3%	1			
**CIV Specific subunits**										
COXEG1	comp54737_c0_seq2	MFS transporter	4a	246	43-246	93.6%	3		1 × PC1, 1 × 3PE	
COXEG2	comp50120_c1_seq1_cut	hypothetical protein [Monosiga brevicollis MX1]	4c	139	16-138	88.5%	1		1 × CDL	
COXEG3	gnl|Egra|Contig2618	hypothetical protein HELRODRAFT_75559	4d	174	2-174	99.4%	1		2 × 3PE, 1 × S12	

COXEG4	EG_transcript_26565	Unknown protein	4e	165	2-161	97.0%	1		1 × PC1	
COXEG5	gnl|Egra|Contig715<br>sga_contig_1482041	Unknown protein	4f	NA	1-75	NA	1			
COXEG6	comp55436_c0_seq3<br>_cut	cytochrome c oxidase assembly<br>Euglenozoa-specific subunit 4	4g	315	19-315	94.3%	0			
COXEG7	gi|109781798_cut	cytochrome oxidase subunit 5	4h	221	17-221	92.8%	0			
COXEG8	comp57506_c0_seq2<br>_cut	Unknown protein	4i	274	10-274	96.7%	0			
COXEG9	comp53374_c0_seq4	Unknown protein	4j	88	1-88	100%	0			

Supplementary Table 5. Eg-CI model summaries

Subunit Name	Subunit identifier	Annotation	Chain ID	# Total residues	Atomic Residues	% Atomic	TMH	Ligands	Lipids	Notes
NDUFV1	comp62912_c0 _seq. 6	NADH dehydrogenase [ubiquinone] flavoprotein 1, mitochondrial	V1	526	18-521	95.8%	0	1 × 4Fe4S, 1 × FMN, 1 × NAI		NAI does not exist in the deactive dataset
NDUFV2	EG_transcript_19472 sga_contig_466476 sga_contig_302717	NADH dehydrogenase [ubiquinone] flavoprotein 2	V2	NA	5-225	NA	0	1 × 2Fe2S		Poly-UNK: 1-4
NDUFS1A	comp61469_c0_seq1 _c	NADH dehydrogenase/NADH:ubiquinone oxidoreductase 75 kDa subunit	1A	385	25-376	91.4%	0	1 × 2Fe2S, 2 × 4Fe4S, 1 × K		
NDUFS1B	EG_transcript_7035	16S rRNA (cytosine(1402)-N(4))-methyltransferase	1B	527	3-527	99.6%	0			
NDUFS2	EG_transcript_13592	NADH dehydrogenase subunit D; NADH:ubiquinone oxidoreductase 49 kDa subunit 7	S2	395	2-395	99.7%	0			N3,N4-dimethylarginne
NDUFS3	comp62960_c0 _seq11	NADH dehydrogenase subunit C	S3	277	26-273	89.5%	0			
NDUFS7	EG_transcript_32395	NADH dehydrogenase [ubiquinone] Fe-S protein 7	S7	NA	7-202	NA	0	1 × 4Fe4S		Poly-UNK: 203-207
NDUFS8	comp54309_c0_seq4 _c	NADH dehydrogenase subunit I	S8	212	30-211	85.8%	0	2 × 4Fe4S		
ND1	sga_contig_684056	NADH-ubiquinone oxidoreductase chain 1	N1	670	361-670	46.3%	8		2 × PC1	

ND2A	sga_contig_881214	Ymf65	N2	300	4-299	98.7%	11		1 × PC1	
ND2B	sga_contig_1967065	NADH dehydrogenase subunit 2	2B	NA	3-115	NA	3			Poly-UNK: 116-142
ND3	sga_contig_1876491	NADH-ubiquinone oxidoreductase chain 3	N3	NA	172-292	NA	3		1 × PC1	
ND4	sga_contig_171091	NADH-ubiquinone oxidoreductase chain 4L	N4	478	1-478	100%	14	1×U10	1 × CDL, 2 × PC1, 1 ×3PE	
ND4L	EG_transcript_32304	NADH dehydrogenase subunit 5	4L	171	61-168	63.2%	3			
ND5	sga_contig_1876491	NADH dehydrogenase subunit 5	N5	584	1-584	100%	15		3 × PC1, 2 × CDL, 1 × 3PE	
ND6	sga_contig_1876491	ADH-ubiquinone oxidoreductase chain 6	N6	NA	18-171	NA	5			
NDUFA2	EG_transcript_28989	NADH dehydrogenase [ubiquinone] 1 alpha subcomplex subunit 2	A2	193	2-193	99.5%	0			
NDUFA5	EG_transcript_24541	NADH dehydrogenase [ubiquinone] 1 alpha subcomplex subunit 5	A5	184	21-174	83.7%	0			
NDUFA6	EG_transcript_12102	NADH dehydrogenase [ubiquinone] 1 alpha subcomplex subunit 6	A6	434	12-434	97.5%	0			
NDUFA7	EG_transcript_32205	NADH dehydrogenase [ubiquinone] 1 alpha subcomplex subunit 7	A7	136	1-136	100%	0			

NDUFA9	comp54702_c0_seq3	NADH dehydrogenase [ubiquinone] 1 alpha subcomplex subunit 9	A9	489	6-489	99.0%	0	1 × NDP	2 × PC1	
NDUFA12	EG_transcript_19840	NADH dehydrogenase [ubiquinone] 1 alpha subcomplex subunit 12	AL	281	17-281	94.3%	0		1 × PC1, 3 × CDL	
NDUFS4	EG_transcript_30365	NADH dehydrogenase [ubiquinone] iron-sulfur protein 4	S4	NA	16-205	NA	0			
NDUFS6	comp55416_c0_seq4	NADH dehydrogenase [ubiquinone] iron-sulfur protein 6	S6	147	1-147	100%	0	1 × Zn^{2+}		
NDUEG1	comp63840_c0_seq1	Adenylate forming domain, Class I	E1	NA	26-475	NA	0			
NDUEG2	sga_contig_620624 EG_transcript_10882	NADH dehydrogenase [ubiquinone] Euglenozoa-specific subunit 12	E2	NA	2-467	NA	0			
NDUEG3	comp63125_c0_seq1	2-enoyl thioester reductase (N,M)	E3	433	2-433	99.8%	0			
NDUEG4	comp59654_c0_seq4	NADH dehydrogenase [ubiquinone] subunit	E4	368	18-368	95.4%	0		1 × PC1	
NDUEG5	EG_transcript_18307	oxidoreductase, putative	E5	289	1-29/32-75/79-234/238-289	96.9%	0			
NDUEG6	comp60945_c0_seq5_c	DnaJ molecular chaperone homology domain	E6	371	30-303/308-371 (NADH)<br>30-371 (turnover)	91.1%<br>92.2%	0		1 × CDL	

					30-303/308-371 (deactive)	91.1%				
NDUEG12	EG_transcript_41155	NADH dehydrogenase [ubiquinone] 1 alpha/beta subcomplex 1	EC	101	17-101	84.2%	0			
NDUFA1	comp54200_c0_seq1	NADH dehydrogenase [ubiquinone] 1 alpha subcomplex subunit 1	A1	141	2-138	97.2%	1		2 × PC1	
NDUFA3	comp51611_c0_seq1_cut	NADH dehydrogenase [ubiquinone] 1 alpha subcomplex subunit 3	A3	125	1-124	99.2%	1		1 × CDL	
NDUFA8	EG_transcript_27781	NADH dehydrogenase [ubiquinone] 1 alpha subcomplex subunit 8	A8	223	1-223	100%	0			
NDUFA11	comp58177_c0_seq3	NADH dehydrogenase [ubiquinone] 1 alpha subcomplex subunit 11	AN	287	1-287	100%	5		1 × 3PE, 1 × PC1	
NDUFA13	comp59406_c0_seq3	NADH dehydrogenase [ubiquinone] 1 alpha subcomplex subunit 13	AM	198	15-198	92.9%	1		3 × CDL, 2 × PC1	
NDUFB2	comp54442_c0_seq1	NADH dehydrogenase [ubiquinone] 1 beta subcomplex subunit 2	B2	NA	41-145	NA	1			
NDUFB3	sga_contig_549447	NADH dehydrogenase [ubiquinone] 1 beta subcomplex subunit 3	B3	NA	2-34	NA	1		1 × CDL	Poly-UNK: 35-62

NDUFB4	EG_transcript_36579	NADH dehydrogenase [ubiquinone] 1 beta subcomplex subunit 4	B4	171	1-171	100%	1			
NDUFB5	EG_transcript_44892	NADH dehydrogenase [ubiquinone] 1 beta subcomplex subunit 5	B5	NA	1-36/43-140	NA	1		2 × PC1, 1 × CDL	Poly-UNK: 37-42
NDUFB6	sga_contig_1395648 sga_contig_881622	NADH dehydrogenase [ubiquinone] 1 beta subcomplex subunit 6	B6	NA	1-91	NA	1			
NDUFB7	sga_contig_1444062	NADH dehydrogenase [ubiquinone] 1 beta subcomplex subunit 7	B7	NA	1-97	NA	0			
NDUFB8	EG_transcript_27273	NADH dehydrogenase [ubiquinone] 1 beta subcomplex subunit 8	B8	176	25-171	83.5%	1			
NDUFB9	comp53986_c0_seq1_cut	NADH dehydrogenase [ubiquinone] 1 beta subcomplex subunit 9	B9	158	8-158	95.6%	0			
NDUFB10	comp54566_c0_seq2	NADH dehydrogenase [ubiquinone] 1 beta subcomplex subunit 10	BL	144	1-144	100%	0			
NDUFB11	comp51117_c0_seq1 sga_contig_881622	NADH dehydrogenase [ubiquinone] 1 beta subcomplex subunit 11	BM	NA	1-100	NA	1			Poly-UNK: 101-112
NDUFC2	comp52747_c0_seq1_cut	NADH dehydrogenase [ubiquinone] 1 subunit C2	C4	185	1-183	98.9%	1		2 × CDL, 1 × PC1	
NDUFAB1-α	gnl|Egra|Contig2592	Acyl carrier protein, mitochondrial	AB	134	47-134	65.7%	0	1 × ZMP		

NDUFAB1-β	gnl|Egra|Contig2076	Acyl carrier protein, mitochondrial	AC	134	43-134	68.7%	0	1 × ZMP		
NDUFS5	EG_transcript_58467	NADH dehydrogenase [ubiquinone] iron-sulfur protein 5	S5	122	1-111	91.0%	0			Poly-UNK: 112-122
NDUFX	comp52123_c0_seq2_gi|125990644	Glyceraldehyde 3-phosphate dehydrogenase, C-terminal domain	FX	325	88-324	72.9%	0			
NDUCA1	comp62122_c0_seq3_c	NADH dehydrogenase [ubiquinone] subunit; CA3 (gamma carbonic anhydrase)	G1	436	34-436 (NADH)	92.4%	0		1 × 3PE	
					11-436 (turnover)	97.7%				
					34-436 (deactive)	92.4%				
NDUCA2	comp48089_c0_seq3_cut	NADH dehydrogenase [ubiquinone] subunit; CA3 (gamma carbonic anhydrase)	G2	267	2-237	88.4%	0			
NDUCA3	EG_transcript_22381 EG_transcript_46696 sga_contig_833122_5'-3' Frame 3	NADH dehydrogenase [ubiquinone] subunit; CA3 (gamma carbonic anhydrase)	G3	NA	8-261	NA	0			Poly-UNK: 1-7
NDUEG7	sga_contig_833122_5'-3' Frame 3 EG_transcript_22716	LRAT-like domain-containing protein	E7	NA	1-246	NA	3	1 × Zn^{2+}	1 × CDL	
NDUEG8	comp47716_c0_seq2_cut	hypothetical protein BRAFLDRAFT_83552	E8	205	1-205	100%	1		4 × PC1	
NDUEG10	EG_transcript_65450	Alpha-2-antiplasmin	EA	126	3-99	77.0%	1		2 × CDL	Poly-UNK: 100-126
NDUEG11	sga_contig_1835241 sga_contig_1398522	Unknown protein	EB	NA	28-101	NA	0			Poly-UNK: 1-27

NDUEG13	comp41364_c0_seq3	Unknown protein	ED	151	14-151	91.4%	1		1 × PC1	
U1	-	-	U1	NA	1-12	NA	0			Poly-UNK: 1-12
U2	-	-	U2	NA	1-12	NA	0			Poly-UNK: 1-12

Supplementary Table 6. Subunit conservation of CI with known structures

Core (green), conserved (blue), partially conserved (yellow) and lineage-specific (no color) subunits of CI are highlighted by different colors. Split core subunits are highlighted pale orange.

**Domain**	**Bacteria**		**Eukaryota**					
**Supergroup**			Opisthokonta		Archaeplastida		Alveolata	Discoba
	Enterobacteria	Deinococcus Thermus	Fungi	Metazoan	Plantae	Chlorophyta	Ciliophora	Euglenozoa
**Species**	*Escherichia coli*	*Thermus Thermophilus*	*Yarrowia lipolytica*	*Ovis aries*	*Arabidopsis thaliana*	*Polytomella*	*Tetrahymena thermophila*	*Euglena gracilis*
**Peripheral Arm Core**	NuoF	Nqo1	NUBM	NDUFV1	NDUV1	NDUV1	NDUV1	NDUV1
	NuoE	Nqo2	NUHM	NDUFV2	NDUV2	NDUV2	NDUV2	NDUV2
	NuoG	Nqo3	NUAM	NDUFS1	NDUS1	NDUS1	NDUS1	NDUS1A
								NDUS1B
	NuoCD	Nqo4	NUCM	NDUFS2	NAD7	NAD7	NDUS2	NDUS2
		Nqo5	NUGM	NDUFS3	NAD9	NAD9	NDUS3	NDUS3
	NuoI	Nqo9	NUIM	NDUFS8	NDUS8	NDUS8	NDUS8	NDUS8
	NuoB	Nqo6	NUKM	NDUFS7	NDUS7	NDUS7	NDUS7	NDUS7
**Membrane Arm Core**	NuoH	Nqo	NU1M	ND1	NAD1	NAD1	ND1A	ND1
							ND1B	
	NuoA	Nqo	NU3M	ND3	NAD3	NAD3	ND3	ND3
	NuoJ	Nqo	NU6M	ND6	NA6	NA6	ND6	ND6
	NuoK	Nqo	NULM	ND4L	NAD4L	NAD4L	ND4L	ND4L
	NuoN	Nqo	NU2M	ND2(11TMH)	NAD2	NAD2	ND2A	ND2a
							ND2B	ND2b
	NuoM	Nqo	NU4M	ND4	NAD4	NAD4	ND4	ND4
	NuoL	Nqo	NU5M	ND5	NAD5	NAD5	ND5A	ND5
							ND5B	
**Conserved Accessory Subunits**			NIMM	NDUFA1	NDUA1	NDUA1	NDUA1	NDUA1
			NI8M	NDUFA2	NDUA2	NDUA2	NDUA2	NDUA2
			NI9M	NDUFA3	NDUA3	NDUA3	NDUA3	NDUA3
			NUFM	NDUFA5	NDUA5	NDUA5	NDUA5	NDUA5
			NB4M	NDUFA6	NDUA6	NDUA6	NDUA6	NDUA6
			NUZM	NDUFA7	NDUA7	NDUA7	NDUA7	NDUA7
			NUPM	NDUFA8	NDUA8	NDUA8	NDUA8	NDUA8
			NUEM	NDUFA9	NDUA9	NDUA9	NDUA9	NDUA9
			NUJM	NDUFA11	NDUA11	NDUA11	NDUA11	NDUA11
			N7BM	NDUFA12	NDUA12	NDUA12	NDUA12	NDUA12
			NB6M	NDUFA13	NDUA13	NDUA13	NDUA13	NDUA13
			NIGM	NDUFB2	NDUB2	NDUB2	NDUB2	NDUB2
			NB2M	NDUFB3	NDUB3	NDUB3	NDUB3	NDUB3
			NB5M	NDUFB4	NDUB4	NDUB4	NDUB4	NDUB4
			NUUM	NDUFB6	NDUB6	NDUB6	NDUB6	NDUB6
			NB8M	NDUFB7	NDUB7	NDUB7	NDUB7	NDUB7
			NIAM	NDUFB8	NDUB8	NDUB8	NDUB8	NDUB8
			NI2M	NDUFB9	NDUB9	NDUB9	NDUB9	NDUB9
			NIDM	NDUFB10	NDUB10	NDUB10	NDUB10	NDUB10
			NESM	NDUFB11	NDUB11	NDUB11	NDUB11	NDUB11
			NEBM	NDUFC2	NDUC2	NDUC2	NDUC2	NDUC2
			ACMP1	NDUFAB1-α	NDUAB1-α	NDUAB1-α	NDUAB1-α	NDUAB1-α
			ACMP2	NDUFAB1-β	NDUAB1-β	NDUAB1-β	NDUAB1-β	NDUAB1-β
			NUYM	NDUFS4	NDUS4	NDUS4	NDUS4	NDUS4
			NIPM	NDUFS5	NDUS5	NDUS5	NDUS5	NDUS5
			NUMM	NDUFS6	NDUS6	NDUS6	NDUS6	NDUS6
**Partially Conserved Accessory Subunits**			NUUM	NDUFB5	NDUP1	NUOP5		
					NDUP9	NDUP9		
			NUXM		NDUX1	NDUX1	NDUX1	
					gCA1	gCA1	NDUCA1	NDUCA1
					gCA2	gCA2	NDUCA2	NDUCA2
					gCAL2	gCAL2	NDUCA3	NDUCA3
					CI-FX	CI-FX	NDUFX	NDUFX
							NDUTT2	NDUEG1
**Lineage Specific Accessory Subunits**		Nqo15	ST1	NDUFA10	NDUP2	NUOP4	NDUTT1	NDUEG2
		Nqo16		NDUFB1		NUOP7	NDUTT3	NDUEG3
				NDUFC1		NUOP8	NDUTT4	NDUEG4
						Unknown sub	NDUTT5	NDUEG5
							NDUTT6	NDUEG6
							NDUTT7	NDUEG7
							NDUTT8	NDUEG8
							NDUTT9	NDUEG9
							NDUTT10	NDUEG10
							NDUTT11	NDUEG11
							NDUTT12	NDUEG12
							NDUTT13	NDUEG13

										NDUTT14	Unknown sub1
										NDUTT15	Unknown sub2
										NDUTT16	
										NDUTT17	
										NDUJ1	
										NDUTX	
										NDUPH1	
										NDUPH2	

Supplementary Table 7. Subunit conservation of $CIII_2$ with known structures

Core (green), conserved (blue), partially conserved (yellow) and lineage-specific (no color) subunits of $CIII_2$ are highlighted by different colors.

**Domain**	**Bacteria**			**Eukaryota**						
**Supergroup**				Opisthokonts			Archaeplastida		Alveolata	Discoba
	Rhodobacteraceae			Fungi	Metazoan (Animalia)		Plantae		Ciliophora	Euglenozoa
**Species**	*Paracoccus denitrificans*	*Rhodobactor sphaeroides*		*Saccharomyces cerevisiae*	*Bos taurus*		*Vigna radiata*		*Tetrahymena thermophila*	*Euglena gracilis*
**Core Subunits**	CYTB	CYTB		Cytb	MT-CYB		COB		COB	CO
	CYTC	CYTC		Cytc1	CYC1		CYC1		CYC1	CYC1
	RISP	RIESKE		Rip1	UQCRFS1/ISP		UCR1		UQCRFS1	UQCRFS1
**Conserved Accessory Subunits**				Cor1	UQCRC1/Core1		MPP-β		MPP-β	MPP-β
				Cor2	UQCRC2/Core2		MPP-α		MPP-α	MPP-α
				Qcr7	UQCRB/Sub6		QCR7		UQCRB	UQCRB
				Qcr8	UQCRQ/Sub7		QCR8		UQCRQ	UQCRQ
				Qcr6	UQCRH/Sub8		QCR6		UQCRH	UQCRH
				Qcr9	UQCR10/Sub10		QCR9		UQCR9	UQCR9
				Qcr10	UQCR11/Sub11		QCR10		UQCR10	UQCR10
**Lineage Specific Subunits**					Sub9 (UQCRFS1 NT MTS)				UQCRTT2	UQCREG1
									Unknown sub	

Supplementary Table 8. Subunit conservation of $CIII_2$ with known structures

Core (green), conserved (blue), partially conserved (yellow) and lineage-specific (no color) subunits of CIV are highlighted by different colors. Split core subunits are highlighted pale orange.

**Domain**	**Bacteria**			**Eukaryota**						
**Supergroup**				Opisthokonts			Archaeplastida		Alveolata	Discoba
	Rhodobacteraceae			Fungi	Metazoan (Animalia)		Plantae		Ciliophora	Euglenozoa
**Species**	*Paracoccus denitrificans*	*Rhodobactor sphaeroides*		*Saccharomyces cerevisiae*	*Bos taurus*		*Vigna radiata*		*Tetrahymena thermophila*	*Euglena gracilis*
**Core Subunits**	COXI	COXI		COXI	COXI		COXI		COX1	COX1
	COXII	COXII		COXII	COXII		COXII		COX2	COX2
		COXIII		COXIII	COXIII		COXIII		COX3A/Ymf67	COX3
									COX3B/Ymf68	
**Conserved Accessory Subunits**				COX5B	COX5B		COX5B-2		COX5B	COX5B-2
				COX6A	COX6A		COX6A		COX6A	COX6A
				COX6B	COX6B		COX6B		COX6B	COX6B-1
				COX7A	COX7A		COX7A		COX7A	COX7A
**Partially Conserved Accessory Subunits**				COX4	COX4-1		COX4			COX4
							COX5C			COX5C
					COX6C				COX6C	
					COX7C		COX7C		COX7C	COX7C
				COX8	COX8					
**Lineage Specific Subunits**				COX6	COX5A				COX6BL	COXEG1
				COX7	COX7B				COX17L	COXEG2
				COX26					COXMC1	COXEG3
									COXMC2	COXEG4
									COXMC3	COXEG5
									COXTIM1	COXEG6
									COXTIM2	COXEG7
									COXTIM3	COXEG8
									COXTIM4	COXEG9
									COXTIM5	
									COXTIM6	
									COXBP	
									COXFS	
									COXAC	
									Ymf67	

									Ymf70	
									Ymf75	
									COXTT1	
									COXTT2	
									COXTT3	
									COXTT4	
									COXTT5	
									COXTT6	
									COXTT7	
									COXTT8	
									COXTT9	
									COXTT10	
									COXTT11	
									COXTT12	
									NDUA4/COXTT1 3	
									COXTT14	
									COXTT15	
									COXTT16	
									COXTT17	
									COXTT18	
									COXTT19	
									COXTT20	
									COXTT21	
									COXTT22	
									COXTT23	
									COXTT24	
									COXTT25	
									COXTT26	
									COXTT28	
									Unknown sub1	
									Unknown sub2	

Supplementary Table 9. Reliability and reproducibility for molecular dynamics simulations

Reliability and reproducibility information	Yes	N/A	Where this information can be found in the text
**1. Convergence of simulations and analysis**			
1a. Is an evaluation presented in the text to show that the property being measured has equilibrated in the simulations (*e.g.* time-course analysis)?	☒		Supplementary figure 19
1b. Then, is it described in the text how simulations are split into equilibration and production runs and how much data were analyzed from production runs?	☒		Yes. The details are described in the *Molecular Dynamics Simulation* subsection of the **Methods** section.
1c. Are there at least 3 simulations per simulation condition with statistical analysis?	☒		Yes. For each simulation condition, there are a minimum of three simulations accompanied by statistical analysis. This information can be found in Figure 6 and under the *Molecular Dynamics Simulation* subsection of the **Methods** section.
1d. Is evidence provided in the text that the simulation results presented are independent of initial configuration?	☒		Yes. The text includes Figure 6 and Supplementary figure 19 demonstrating that the simulation outcomes are independent of the initial configuration. This information is detailed in the *Molecular Dynamics Simulation* subsection of the **Methods** section.
**2. Connection to experiments**			
2a. Are calculations provided that can connect to experiments (*e.g.* loss or gain in function from mutagenesis, binding assays, NMR chemical shifts, J-couplings, SAXS curves, interaction distances or FRET distances, structure factors, diffusion coefficients, bulk modulus and other mechanical properties, *etc.*)?	☒		Yes. The calculations include aspects such as membrane curvature around the protein and the binding complex, which can be validated through experimental methods.
**3. Method choice**			
3a. Is it described in the text what force field and water model are used and why?	☒		Yes. We used CHARMM36m and Martini3 force fields as described in the *Molecular Dynamics Simulation* subsection of the **Methods** section.
3b. Do simulations contain membranes, membrane proteins, intrinsically disordered proteins, glycans, nucleic acids, polymers, or cryptic ligand binding?	☒	☐	
If 3b is **YES**, are enhanced sampling methods used?	☒	☐	Yes. We used coarse-grained Martini3 model to enhance the sampling.

If enhanced sampling methods are used, are the convergence criteria clearly stated?	☒		Yes. The criteria for convergence are clearly outlined in the **Methods** section in the context of our utilization of enhanced sampling methods.
If 3b is **YES**, is it explained in the text why or why not enhanced sampling methods are used?	☒		Yes. The text provides an explanation for our choice to use enhanced sampling methods, specifically mentioning the implementation of coarse-grained simulations for this purpose.
**4. Code and reproducibility**			
4a. Is a table provided describing the system setup, such as simulation box dimensions, total number of atoms, total number of water molecules, salt concentration, lipid composition (number of molecules and type)?	☐		No. The system setup details are not presented in a table format. Instead, they are thoroughly described in the **Methods** section.
4b. Is it described in the text what simulation and analysis software and which versions are used?	☒		Yes. In **Methods** section, we specify the simulation and analysis software employed, along with their respective versions.
4c. Are initial coordinate and simulation input files and a coordinate file of the final output provided as supplementary files or in a public repository?	☒		Yes. The files have been uploaded to GitHub and are accessible via the following link: https://github.com/yongwangCPH/papers/tree/main/2024/CIV-CytC
4d. Is there custom code or custom force field parameters?	☐	☒	
If **YES**, are they provided as supplementary profiles or in a public repository?	☐		

Supplementary References

1. Airenne, T. T. *u.c.* Structure-function analysis of enoyl thioester reductase involved in mitochondrial maintenance. *Journal of molecular biology* **327**, 47–59 (2003).
2. Wierenga, R. K., Terpstra, P. & Hol, W. G. J. Prediction of the occurrence of the ADP-binding beta alpha beta-fold in proteins, using an amino acid sequence fingerprint. *Journal of molecular biology* **187**, 101–7 (1986).
3. Meijers, R. *u.c.* On the enzymatic activation of NADH. *The Journal of biological chemistry* **276**, 9316–21 (2001).
4. Hoffmeister, M., Piotrowski, M., Nowitzki, U. & Martin, W. Mitochondrial trans-2-enoyl-CoA reductase of wax ester fermentation from Euglena gracilis defines a new family of enzymes involved in lipid synthesis. *The Journal of biological chemistry*

280, 4329–38 (2005).

5. Tomiyama, T. *u.c.* A major isoform of mitochondrial trans-2-enoyl-CoA reductase is dispensable for wax ester production in Euglena gracilis under anaerobic conditions. *PloS one* **14**, e0210755 (2019).
6. Nakazawa, M. *u.c.* Anaerobic respiration coupled with mitochondrial fatty acid synthesis in wax ester fermentation by Euglena gracilis. *FEBS letters* **592**, 4020–4027 (2018).
7. Tari, L. W., Matte, A., Goldie, H. & Delbaere, L. T. J. Mg(2+)-Mn2+ clusters in enzyme-catalyzed phosphoryl-transfer reactions. *Nature structural biology* **4**, 990–4 (1997).
8. Trapani, S. *u.c.* Crystal structure of the dimeric phosphoenolpyruvate carboxykinase (PEPCK) from Trypanosoma cruzi at 2 A resolution. *Journal of molecular biology* **313**, 1059–72 (2001).
9. Andersson, C. S. *u.c.* The Mycobacterium tuberculosis very-long-chain fatty acyl-CoA synthetase: structural basis for housing lipid substrates longer than the enzyme. *Structure (London, England : 1993)* **20**, 1062–70 (2012).
10. Goyal, A., Verma, P., Anandhakrishnan, M., Gokhale, R. S. & Sankaranarayanan, R. Molecular basis of the functional divergence of fatty acyl-AMP ligase biosynthetic enzymes of Mycobacterium tuberculosis. *Journal of molecular biology* **416**, 221–38 (2012).
11. Zhou, L., Maldonado, M., Padavannil, A., Guo, F. & Letts, J. A. Structures of Tetrahymena's respiratory chain reveal the diversity of eukaryotic core metabolism. *Science (New York, N.Y.)* **376**, 831–839 (2022).
12. Padavannil, A., Ayala-Hernandez, M. G., Castellanos-Silva, E. A. & Letts, J. A. The Mysterious Multitude: Structural Perspective on the Accessory Subunits of Respiratory Complex I. *Frontiers in molecular biosciences* **8**, 798353 (2021).
13. Gu, J., Liu, T., Guo, R., Zhang, L. & Yang, M. The coupling mechanism of mammalian mitochondrial complex I. *Nature structural & molecular biology* **29**, 172–182 (2022).
14. Kampjut, D. & Sazanov, L. A. The coupling mechanism of mammalian respiratory

complex I. *Science (New York, N.Y.)* **370**, e0227226 (2020).
15. Pereira, C. S., Teixeira, M. H., Russell, D. A., Hirst, J. & Arantes, G. M. Mechanism of rotenone binding to respiratory complex I depends on ligand flexibility. *Scientific reports* **13**, 6738 (2023).
16. Wu, M., Gu, J., Guo, R., Huang, Y. & Yang, M. Structure of Mammalian Respiratory Supercomplex I1III2IV1. *Cell* **167**, 1598-1609.e10 (2016).
17. Letts, J. A., Fiedorczuk, K. & Sazanov, L. A. The architecture of respiratory supercomplexes. *Nature* **537**, 644–648 (2016).
18. Pei, J. & Grishin, N. V. The Rho GTPase inactivation domain in Vibrio cholerae MARTX toxin has a circularly permuted papain-like thiol protease fold. *Proteins* **77**, 413–9 (2009).
19. Shimada, S. *u.c.* Complex structure of cytochrome c-cytochrome c oxidase reveals a novel protein-protein interaction mode. *The EMBO journal* **36**, 291–300 (2017).
20. Miranda-Astudillo, H. V. *u.c.* The atypical subunit composition of respiratory complexes I and IV is associated with original extra structural domains in Euglena gracilis. *Scientific reports* **8**, 9698 (2018).
21. Vercellino, I. & Sazanov, L. A. Structure and assembly of the mammalian mitochondrial supercomplex CIII2CIV. *Nature* **598**, 364–367 (2021).
22. Fang, H. *u.c.* A membrane arm of mitochondrial complex I sufficient to promote respirasome formation. *Cell reports* **35**, 108963 (2021).
23. Miranda-Astudillo, H. V., Yadav, K. N. S., Boekema, E. J. & Cardol, P. Supramolecular associations between atypical oxidative phosphorylation complexes of Euglena gracilis. *Journal of bioenergetics and biomembranes* (2021). doi:10.1007/s10863-021-09882-8
24. Han, F. *u.c.* Structures of Tetrahymena thermophila respiratory megacomplexes on the tubular mitochondrial cristae. *Nature communications* **14**, 2542 (2023).
25. Mühleip, A. *u.c.* Structural basis of mitochondrial membrane bending by the I-II-III2-IV2 supercomplex. *Nature* **615**, 934–938 (2023).
26. Archibald, J. M., Simpson, A. G. B. & Slamovits, C. H. *Handbook of the Protists. Handbook_of_Protists* (Springer International Publishing, 2017). doi:10.1007/978-3-

319-28149-0

27. Mühleip, A. W., Dewar, C. E., Schnaufer, A., Kühlbrandt, W. & Davies, K. M. In situ structure of trypanosomal ATP synthase dimer reveals a unique arrangement of catalytic subunits. *Proceedings of the National Academy of Sciences of the United States of America* **114**, 992–997 (2017).
