# Supplementary material for: Euglena’s atypical respiratory chain adapts to the discoidal cristae and flexible metabolism
Source: Nat Commun. 2024 Feb 22;15:1628. doi: 10.1038/s41467-024-46018-z (PMC10884005; doi:10.1038/s41467-024-46018-z)
Supplement: Supplementary file 3 — Description of Additional Supplementary Files [file 41467_2024_46018_MOESM3_ESM.pdf]

Description of Additional Supplementary Files

File name: Supplementary Movie 1
Description: CryoEM density map and model for E. gracilis SC I+III_2+IV.

File name: Supplementary Movie 2
Description: CryoEM density map and model for E. gracilis SC III_2+IV_2.

File name: Supplementary Movie 3
Description: Swinging motions of the $CIII_2$ Rieske heads in E. gracilis SC I+III2+IV generated by 3DVA.

File name: Supplementary Movie 4
Description: Swinging motions of the $CIII_2$ Rieske heads in E. gracilis SC III_2+IV_2 generated by 3DVA.

File name: Supplementary Movie 5
Description: Coarse-grained molecular dynamics simulation of Eg-SC I+III_2+IV in lipid bilayer.

File name: Supplementary Movie 6
Description: Coarse-grained molecular dynamics simulation of Tt-SC I+III_2 in lipid bilayer
