# Supplementary material for: Euglena’s atypical respiratory chain adapts to the discoidal cristae and flexible metabolism
Source: Nat Commun. 2024 Feb 22;15:1628. doi: 10.1038/s41467-024-46018-z (PMC10884005; doi:10.1038/s41467-024-46018-z)
Supplement: Supplementary file 10 — Reporting Summary [file 41467_2024_46018_MOESM10_ESM.pdf]

Corresponding author(s): Long Zhou
Last updated by author(s): Jul 14, 2023

Reporting Summary

Nature Portfolio wishes to improve the reproducibility of the work that we publish. This form provides structure for consistency and transparency in reporting. For further information on Nature Portfolio policies, see our Editorial Policies and the Editorial Policy Checklist.

Statistics

For all statistical analyses, confirm that the following items are present in the figure legend, table legend, main text, or Methods section.

n/a	Confirmed	
☐	☒	The exact sample size (*n*) for each experimental group/condition, given as a discrete number and unit of measurement
☐	☒	A statement on whether measurements were taken from distinct samples or whether the same sample was measured repeatedly
☐	☒	The statistical test(s) used AND whether they are one- or two-sided<br>*Only common tests should be described solely by name; describe more complex techniques in the Methods section.*
☒	☐	A description of all covariates tested
☒	☐	A description of any assumptions or corrections, such as tests of normality and adjustment for multiple comparisons
☐	☒	A full description of the statistical parameters including central tendency (e.g. means) or other basic estimates (e.g. regression coefficient) AND variation (e.g. standard deviation) or associated estimates of uncertainty (e.g. confidence intervals)
☐	☒	For null hypothesis testing, the test statistic (e.g. *F*, *t*, *r*) with confidence intervals, effect sizes, degrees of freedom and *P* value noted<br>*Give P values as exact values whenever suitable.*
☒	☐	For Bayesian analysis, information on the choice of priors and Markov chain Monte Carlo settings
☒	☐	For hierarchical and complex designs, identification of the appropriate level for tests and full reporting of outcomes
☒	☐	Estimates of effect sizes (e.g. Cohen's *d*, Pearson's *r*), indicating how they were calculated

Our web collection on statistics for biologists contains articles on many of the points above.

Software and code

Policy information about availability of computer code

Data collection	EPU 2
Data analysis	ChimeraX 1.4, Coot 0.9.6., CryoSPRAC 3.3.2, Phenix 1.20.1, Relion 4.0, CTFFIND4.1, crYOLO1.6.0, PyMOL 2.5.2, Prism9.3.1, BLAST web server, Phyre2 web server, PredictProtein web server, AlphaFold2, OPM webserver, CHARMM-GUI webserver, CHARMM36, PLUMED, Martini3, Gromacs 2022.5

For manuscripts utilizing custom algorithms or software that are central to the research but not yet described in published literature, software must be made available to editors and reviewers. We strongly encourage code deposition in a community repository (e.g. GitHub). See the Nature Portfolio guidelines for submitting code & software for further information.

Data

Policy information about availability of data

All manuscripts must include a data availability statement. This statement should provide the following information, where applicable:

- Accession codes, unique identifiers, or web links for publicly available datasets
- A description of any restrictions on data availability
- For clinical datasets or third party data, please ensure that the statement adheres to our policy

The composite maps and models for Eg-SC I+III2+IV, Eg-SC III2+IV2 and Eg-CI are available in the Electron Microscopy Database (EMDB) and the Protein Data Bank (PDB) with accession codes as following: Eg-SC I+III2+IV EMDB-35720 (https://www.ebi.ac.uk/emdb/EMD-35720), PDB-8IUF (https://www.rcsb.org/structure/8IUF);

Eg-SC III2+IV2 EMDB-35723 (https://www.ebi.ac.uk/emdb/EMD-35723), PDB-8IUJ (https://www.rcsb.org/structure/8IUJ); Eg-CI turnover EMDB-36108 (https://www.ebi.ac.uk/emdb/EMD-36108), PDB-8J9I (https://www.rcsb.org/structure/8J9I); Eg-CI NADH reduced EMDB-36109 (https://www.ebi.ac.uk/emdb/EMD-36109), PDB-8J9J (https://www.rcsb.org/structure/8J9J); Eg-CI deactivated EMDB-36107 (https://www.ebi.ac.uk/emdb/EMD-36107), PDB-8J9H (https://www.rcsb.org/structure/8J9H).

Consensus and local refinement maps for Eg-SC I+III2+IV, Eg-SC III2+IV2 and Eg-CI are available in the EMDB with accession codes as following: Eg-SC I+III2+IV consensus map EMDB-35819 (https://www.ebi.ac.uk/emdb/EMD-35819), CI-PA region EMDB-35662 (https://www.ebi.ac.uk/emdb/EMD-35662), CI-MA proximal region EMDB-35663 (https://www.ebi.ac.uk/emdb/EMD-35663), CI-MA distal region EMDB-35664 (https://www.ebi.ac.uk/emdb/EMD-35664), CIII2 region EMDB-35665 (https://www.ebi.ac.uk/emdb/EMD-35665) and CIV region EMDB-35666 (https://www.ebi.ac.uk/emdb/EMD-35666); Eg-SC III2+IV2 consensus map EMDB-35820 (https://www.ebi.ac.uk/emdb/EMD-35820), CIII2 region EMDB-35667 (https://www.ebi.ac.uk/emdb/EMD-35667), two CIV regions EMDB-35668 (https://www.ebi.ac.uk/emdb/EMD-35668) and EMDB-35669 (https://www.ebi.ac.uk/emdb/EMD-35669); Eg-CI turnover state consensus map EMDB-36102 (https://www.ebi.ac.uk/emdb/EMD-36102), CI-PA region EMDB-36099 (https://www.ebi.ac.uk/emdb/EMD-36099), CI-MA proximal region EMDB-36100 (https://www.ebi.ac.uk/emdb/EMD-36100), CI-MA distal region EMDB-36101 (https://www.ebi.ac.uk/emdb/EMD-36101); Eg-CI NADH reduced state consensus map EMDB-36106 (https://www.ebi.ac.uk/emdb/EMD-36106), CI-PA region EMDB-36103 (https://www.ebi.ac.uk/emdb/EMD-36103), CI-MA proximal region EMDB-36104 (https://www.ebi.ac.uk/emdb/EMD-36104), CI-MA distal region EMDB-36105 (https://www.ebi.ac.uk/emdb/EMD-36105); Eg-CI NADH deactivated state consensus map EMDB-36098 (https://www.ebi.ac.uk/emdb/EMD-36098), CI-PA region EMDB-36094 (https://www.ebi.ac.uk/emdb/EMD-36094), CI-MA proximal region EMDB-36096 (https://www.ebi.ac.uk/emdb/EMD-36096), CI-MA distal region EMDB-36097 (https://www.ebi.ac.uk/emdb/EMD-36097).

For model building, Euglena gracilis genome and transcriptome used are obtained from GenBank under accession code GCA_900893395.1 (https://www.ncbi.nlm.nih.gov/datasets/genome/GCA_900893395.1/). Initial structural models used are available in the PDB under accession codes 7AR8 (https://www.rcsb.org/structure/7AR8), 7ARD (https://www.rcsb.org/structure/7AR8), 7JRG (https://www.rcsb.org/structure/7JRG) and 7JRO (https://www.rcsb.org/structure/7JRO).

For MD simulations and data presentation, Euglena gracilis cytochrome c and mitochondrial TER sequences are obtained from Uniprot under accession codes P00076 (https://www.uniprot.org/uniprotkb/P00076/entry) and Q5EU90 (https://www.uniprot.org/uniprotkb/Q5EU90/entry), respectively.

Research involving human participants, their data, or biological material

Policy information about studies with human participants or human data. See also policy information about sex, gender (identity/presentation), and sexual orientation and race, ethnicity and racism.

Reporting on sex and gender	N/A
Reporting on race, ethnicity, or other socially relevant groupings	N/A
Population characteristics	N/A
Recruitment	N/A
Ethics oversight	N/A

Note that full information on the approval of the study protocol must also be provided in the manuscript.

Field-specific reporting

Please select the one below that is the best fit for your research. If you are not sure, read the appropriate sections before making your selection.

☒ Life sciences ☐ Behavioural & social sciences ☐ Ecological, evolutionary & environmental sciences

For a reference copy of the document with all sections, see nature.com/documents/nr-reporting-summary-flat.pdf

Life sciences study design

All studies must disclose on these points even when the disclosure is negative.

Sample size	Estimation of the sample size was mainly based on a previous study https://pubmed.ncbi.nlm.nih.gov/31738165/ and cryo-EM studies of similar protein complexes. The numbers of micrographs and particles in the dataset are presented in Supplementary Fig.4, Fig.5, Table. 1 and Table. 2, which demonstrate that they are sufficient to generate map refinements at 2.69-2.77 Å for Eg-SC I+III2+IV, 2.85-3.14 Å for Eg-SC III2 +IV2, 2.82-2.89 Å for Eg-CI turnover state, 2.93-2.97 Å for Eg-CI NADH-reduced state and 3.00-3.07 Å for Eg-CI deactive state.
Data exclusions	There is no pre-set data exclusion criteria. Manual curation excluded less than 1% raw micrographs from both Datasets due to bad imaging quality or contamination. Particle curation by 2D classification and 3D ab-initio reconstruction were used to further exclude particles that would not contribute meaningfuly to the final map reconstruction due to low signal-to-noise ratio or ice contamination.
Replication	Purification of E. gracilis's ETC complexes were performed at least 10 times in LMNG for Eg-CI and at least 5 times in digitonin for Eg-SC I+III2 +IV amd SC III2+IV2, results form which agreed unon each other as shown in Supplementary Fig.1. Different classification methods were employed to isolate particle subsets of Eg-SC I+III2+IV, Eg-SC III2+IV2, Eg-CI turnover state, Eg-CI NADH-reduced state and Eg-CI deactive state, particle numbers from which were constant. 3D refinements of different kinds (homogenous, non-uniform, local refinements, with or without glooal and local refinements) were performed for at east 20 times for each dataset, which gave reproducible maps in the same resolution

range. Extinction coefficient and activity measurements were performed in triplicates with the same protein sample, where all attempts at replication were successful.

Randomization	Randomization was performed in resolution estimation based on Fourier Shell Correlation method (gold standard), where the dataset was randomly split into two halves and refined independently. This process is performed for each 3D refinement shown in Supplementary Fig.4-Fig.9. For purification experiments, different batches of E. gracilis were cultured under identical conditions were considered equivalents. In molecular dynamics simulations, initial velocities were randomized by employing distinct random seeds.
Blinding	Blinding was not pertormed to the studied sample since it is not a standard procedure in structural biology. Raw micrographs or particle images are not categorical data. Particles are randomly assigned into half-sets for image processing; hence no blinding is applicable. This study did not incude experiments with experimental group allocation and thus no blinding was applied. All experimental samples were prepared and analyzed as described in the manuscript.

Reporting for specific materials, systems and methods

We require information from authors about some types of materials, experimental systems and methods used in many studies. Here, indicate whether each material, system or method listed is relevant to your study. If you are not sure if a list item applies to your research, read the appropriate section before selecting a response.

Materials & experimental systems

n/a	Involved in the study
☒	☐ Antibodies
☐	☒ Eukaryotic cell lines
☒	☐ Palaeontology and archaeology
☒	☐ Animals and other organisms
☒	☐ Clinical data
☒	☐ Dual use research of concern
☒	☐ Plants

Methods

n/a	Involved in the study
☒	☐ ChIP-seq
☒	☐ Flow cytometry
☒	☐ MRI-based neuroimaging

Eukaryotic cell lines

Policy information about cell lines and Sex and Gender in Research

Cell line source(s)	Euglena gracilis cells were obtained from Guangyu Biological Technology Co., LTD, Shanghai, China under the catalogue number GY-D32.
Authentication	No further authentications were performed for this study.
Mycoplasma contamination	N/A
Commonly misidentified lines (See ICLAC register)	N/A
